# Dynamics of camel and human hemoglobin revealed by molecular simulations

**DOI:** 10.1038/s41598-021-04112-y

**Published:** 2022-01-07

**Authors:** Amanat Ali, Soja Saghar Soman, Ranjit Vijayan

**Affiliations:** 1 Department of Biology, College of Science, United Arab Emirates University, P.O. Box 15551, Al Ain, United Arab Emirates; 2 New York University Abu Dhabi, P.O. Box 129188, Abu Dhabi, United Arab Emirates; 3 The Big Data Analytics Center, United Arab Emirates University, P.O. Box 15551, Al Ain, United Arab Emirates; 4 Zayed Center for Health Sciences, United Arab Emirates University, P.O. Box 15551, Al Ain, United Arab Emirates

**Keywords:** Blood proteins, Molecular modelling, Carrier proteins

## Abstract

Hemoglobin is one of the most widely studied proteins genetically, biochemically, and structurally. It is an oxygen carrying tetrameric protein that imparts the characteristic red color to blood. Each chain of hemoglobin harbors a heme group embedded in a hydrophobic pocket. Several studies have investigated structural variations present in mammalian hemoglobin and their functional implications. However, camel hemoglobin has not been thoroughly explored, especially from a structural perspective. Importantly, very little is known about how the heme group interacts with hemoglobin under varying conditions of osmolarity and temperature. Several experimental studies have indicated that the tense (T) state is more stable than the relaxed (R) state of hemoglobin under normal physiological conditions. Despite the fact that R state is less stable than the T state, no extensive structural dynamics studies have been performed to investigate global quaternary transitions of R state hemoglobin under normal physiological conditions. To evaluate this, several 500 ns all-atom molecular dynamics simulations were performed to get a deeper understanding of how camel hemoglobin behaves under stress, which it is normally exposed to, when compared to human hemoglobin. Notably, camel hemoglobin was more stable under physiological stress when compared to human hemoglobin. Additionally, when compared to camel hemoglobin, cofactor-binding regions of hemoglobin also exhibited more fluctuations in human hemoglobin under the conditions studied. Several differences were observed between the residues of camel and human hemoglobin that interacted with heme. Importantly, distal residues His58 of α hemoglobin and His63 of β hemoglobin formed more sustained interactions, especially at higher temperatures, in camel hemoglobin. These residues are important for oxygen binding to hemoglobin. Thus, this work provides insights into how camel and human hemoglobin differ in their interactions under stress.

## Introduction

Hemoglobin protein is abundantly present in red blood cells (RBCs) or erythrocytes. It is an oxygen carrying protein that gives the characteristic red color to blood. Adult vertebrate hemoglobin is composed of four protein chains, two α chains and two β chains. These paralogs were produced as a result of gene duplication^1^. Structurally, hemoglobin chains retain the classic globin fold, which is shared by several proteins. Myoglobin, for example, a monomer, retains the same fold with just 25% sequence identity to hemoglobin.

Each hemoglobin chain harbors a heme group in a hydrophobic pocket. A ferrous ion (Fe^2+^) of the heme group associated with each hemoglobin chain acts as a cofactor for this tetrameric protein. The Fe^2+^ ion is coordinated by four nitrogen atoms of the tetrapyrrole ring of heme. Oxygen (O_2_) molecule is transported in blood by reversibly binding to these Fe^2+^ ions. The hemoglobin binding pocket shields the Fe^2+^ from the solvent. Additionally, the Fe^2+^ ion is also coordinated by a fifth nitrogen atom belonging to a proximal histidine of the hemoglobin chain. Distal histidine residues (α:His58 and β:His63) are located further away from the Fe^2+^ ion, and provide a void for oxygen binding. Studies have indicated that these distal histidine residues play a critical role in modulating the rate of oxygen binding as well as its affinity to hemoglobin^2^. Additionally, the role of distal histidine in stabilizing the bound oxygen has also been reported^3^. O_2_ and carbon monoxide (CO) appear to enter hemoglobin subunits via these distal histidine gates. Therefore, it is vital for O_2_ and CO to reach the active site through migration from solution to the protein. However, known protein structures have not shown the presence of channels for diffusion indicating that protein flexibility is required^4–6^. The role of heme in human hemoglobin has been extensively explored in vitro*, *in vivo and in silico^7,8^. However, the stability of these binding interactions, a comparison of the dynamics of human and other mammalian hemoglobin, as well as its dynamics under stress have not been explored.

**Figure 1:**
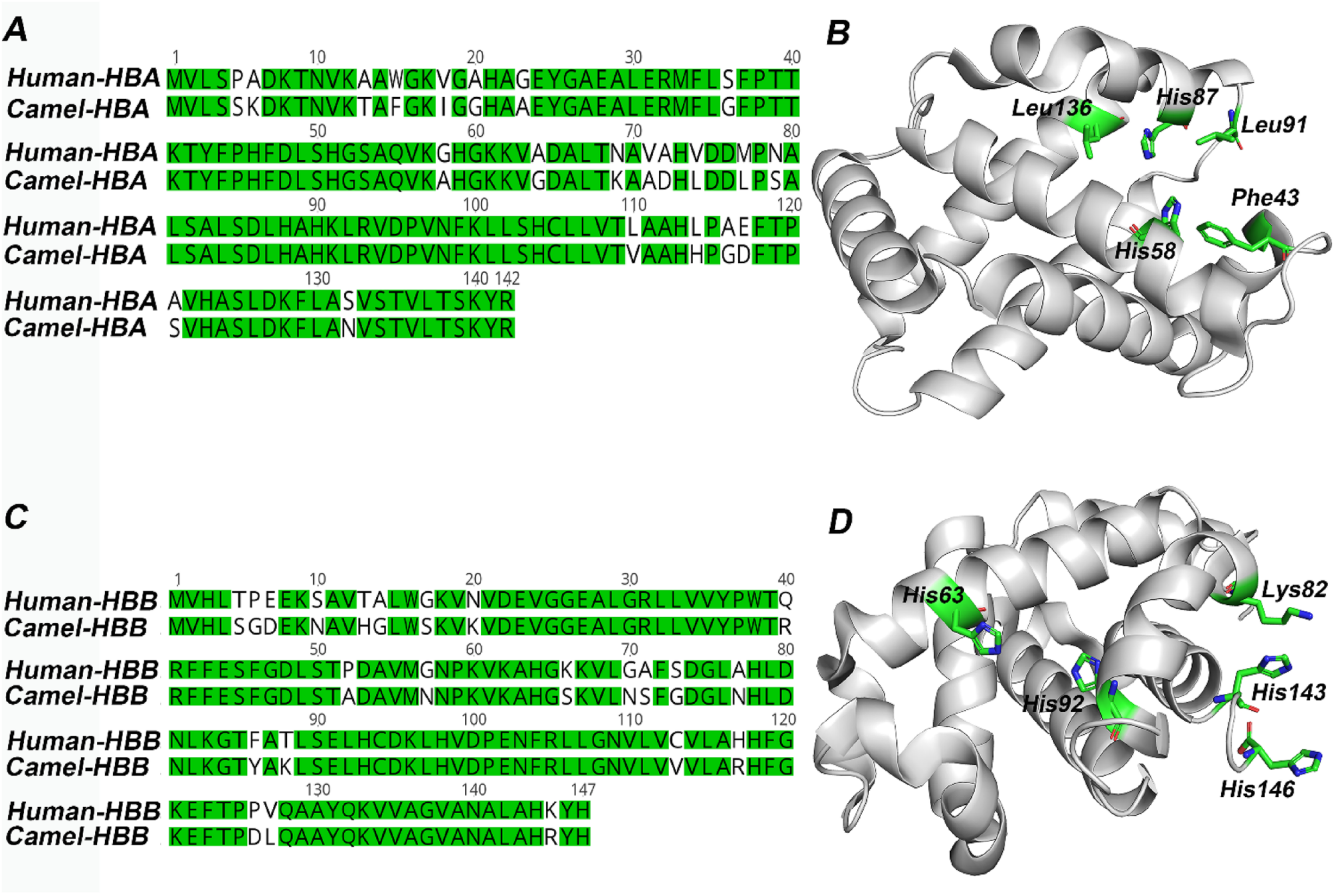
Comparison of camel and human hemoglobin. Sequences were retrieved from UniProt (https://www.uniprot.org) with accession numbers P69905 (human hemoglobin α subunit), P68871 (human hemoglobin β subunit), P63106 (camel hemoglobin α subunit) and P68231 (camel hemoglobin β subunit), and aligned using Geneious 9.1.2 (https://www.geneious.com). (**A**) Sequence alignment of α subunit of camel and human hemoglobin. (**B**) Structure of α subunit of camel hemoglobin (PDB ID: 3GDJ). C) Sequence alignment of β subunit of camel and human hemoglobin. D) Structure of β subunit of camel hemoglobin (PDB ID: 3GDJ). Conserved residues are shown in green. Critical residues of hemoglobin that interact with heme and 2,3-bisphosphoglycerate (2,3-BPG) are shown in green stick representation. Image B and D were generated using Schrödinger Maestro 2019–4 (Schrödinger, LLC, New York, NY).

Camels have unique characteristics that enable them to withstand harsh environmental stresses. Their body temperature could fluctuate between 34 and 41 °C within the day. Additionally, camels are capable of dealing with eight times more salt when compared to other closely related mammals without exhibiting any signs and symptoms of hypertension^9^. Camels have the exceptional ability to live without drinking water for a long period of time. These conditions, in combination, produces a severely dehydrated state in camels. Nearly all other mammals are incapable of withstanding such stress for any significant period of time. Importantly, severe dehydration and high temperature conditions are associated with decreased binding affinity of oxygen molecules^10^. Interestingly, camel hemoglobin contains more charged amino acid residues and are more hydrophilic than the hemoglobin of other mammalian species^11^. The availability of a three-dimensional X-ray crystal structure of the camel hemoglobin molecule now permits structural comparisons of this molecule with that of several other well-studied species including humans, rats and mice^12^. The α and β chains of camel and human hemoglobin share a sequence identity of 85.51% and 84.35%, respectively (Fig. 1). Residues involved in the binding of heme and other ligands such as 2,3-bisphosphoglycerate (2,3-BPG) and adenosine triphosphate (ATP) are conserved in both camel and human hemoglobin. 2,3-BPG and ATP are two important co-factors, present abundantly in the erythrocytes, that bind to hemoglobin^13^. These co-factors assist with the stabilization of the deoxyhemoglobin or tense (T) state of the hemoglobin and are important for unloading O_2_ from hemoglobin in tissues^14,15^.

Indeed, hemoglobin is one of the most well-characterized and widely studied proteins genetically, biochemically, and structurally. Structural and genetic variations among mammalian hemoglobins have been evaluated^16^. However, camel hemoglobin has not been extensively explored. Only a few physiological and biochemical studies have been performed on camel hemoglobin^17,18^. Very little is known about its structure and dynamics. Therefore, a comparative study, principally focused on structural aspects of camel hemoglobin, was undertaken to provide insights into how camel hemoglobin behaves under varying conditions of osmolarity and temperature that it is normally susceptible to. Human hemoglobin was also subjected to equivalent conditions for comparison.

Hemoglobin exists in two important structural states known as the deoxy structure (T or tense) and the oxy structure (R or relaxed). Normally, the T state is stable when a ligand is not bound to the heme–iron, whereas R state is stable when ligands are bound to the heme groups. Molecular dynamics (MD) simulation is a method for probing dynamics and energetics of atoms and molecules with reasonable precision. This, often, complements and assists biological studies. Several promising results have been reported on a variety of biological system^19–22^. All-atom MD simulations have been used to understand the dynamics of T and R states of hemoglobin using different force fields and simulation conditions in the past^23,24^. While recent studies have demonstrated that a large simulation box size is needed to stabilize the T state of hemoglobin^25–28^, the R state has been observed to be stable for long periods of simulation time in smaller simulation boxes^23^. Time-resolved spectroscopic techniques have shown that R → T quaternary transition typically occurs over a period of 21 µs^29^. Despite the fact that R state hemoglobin is less stable than the T state hemoglobin, no extended MD simulations have been performed to study quaternary transitions of this state.

Considering the inherent ability of camel to withstand severe dehydrated conditions compared to other species, we evaluate the effect of different stresses on hemoglobin. We also probe the R state dynamics of both camel and human hemoglobin, in terms of interaction stability of heme molecule with critical residues of hemoglobin, under physiologically normal and induced salt and thermal stress.

## Methods

Three-dimensional X-ray crystal structures of camel and human hemoglobin were obtained from the Protein Data Bank (PDB; https://www.rcsb.org). The PDB IDs of the structures used are 1BBB (R2 state)^30^ for human hemoglobin and 3GDJ (likely R2 as there is no accompanying publication) for camel hemoglobin. The structures were visualized and prepared for simulations using Schrödinger Maestro 2019-4 (Schrödinger, LLC, New York, NY). Structures were pre-processed using the Protein Preparation Wizard (Schrödinger, LLC, New York, NY) to assign partial charges and to complete missing atoms and side chains. The terminal histidines (His146) of β chains were doubly protonated while the protonation states of remaining histidines were kept as described in previous studies^23,25,31^ (Supplementary Table S1). Heme Fe^2+^ ligands were retained during protein preparation. Structures of camel and human hemoglobin were placed in orthorhombic boxes of size 100 Å × 100 Å × 100 Å and/or 150 Å × 150 Å × 150 Å and solvated with single point charge (SPC) water molecules using the Desmond System Builder (Schrödinger, LLC, New York, NY). Counterions were added to neutralize the prepared systems. Systems were prepared with different concentrations—0 mM, 150 mM, 300 mM, and 600 mM—of NaCl at 27 °C. For evaluating the effect of temperature, simulations were run at 27 °C, 30 °C, 34 °C and 41 °C while maintaining a NaCl concentration of 150 mM. Thus, 14 different systems were prepared (Table 1). Desmond was used to perform MD simulations^32^. Molecules and their interactions were described using the OPLS forcefield. Before the production runs, all systems were subjected to Desmond’s default eight stage relaxation protocol. 100 Å box size systems were simulated for a duration of 500 ns, while 150 Å box size systems were simulated for 200 ns. A pressure of 1 atm was maintained using the Martyna–Tobias–Klein barostat^33^ and a constant temperature was maintained using the Nosé–Hoover thermostat^34^. For electrostatic interactions, a short-range cutoff of 9.0 Å was used and the smooth particle mesh Ewald method (PME) was employed for computing long-range coulombic interactions^35^. A time-reversible reference system propagator algorithm (RESPA) integrator was used with an inner time step of 2.0 fs and an outer time step of 6.0 fs^36^. Simulation data was analyzed using various packaged and in-house scripts. Secondary structure of the proteins was monitored, structural stability was evaluated based on root mean square deviation (RMSD) from the initial structure, and positional fluctuations were assessed using root mean square fluctuation (RMSF) of each protein residue. Molecular mechanics—generalized Born surface area (MM-GBSA) method was employed to compute the free energy of binding (ΔG_bind_) of heme to camel and human hemoglobin using frames extracted from MD simulation trajectories. From each simulation, frames were retrieved every 100 ns and MM-GBSA based ΔG_bind_ was estimated using Schrödinger Prime employing the VSGB 2.0 solvation model^37^.

**Table 1:** Temperature and salt conditions used for MD simulations of camel and human hemoglobin.

Organism (PDB ID)	Temperature (°C)	Salt concentration (mM)	Box size
Human (1BBB)	27	0	100 Å
	27	150	100 Å
	27	300	100 Å
	27	600	100 Å and 150 Å
	30	150	100 Å and 150 Å
	34	150	100 Å
	41	150	100 Å
Camel (3GDJ)	27	0	100 Å
	27	150	100 Å
	27	300	100 Å
	27	600	100 Å and 150 Å
	30	150	100 Å and 150 Å
	34	150	100 Å
	41	150	100 Å

## Results

### Evaluation of R → T transition at different salt and temperature conditions

**Figure 2:**
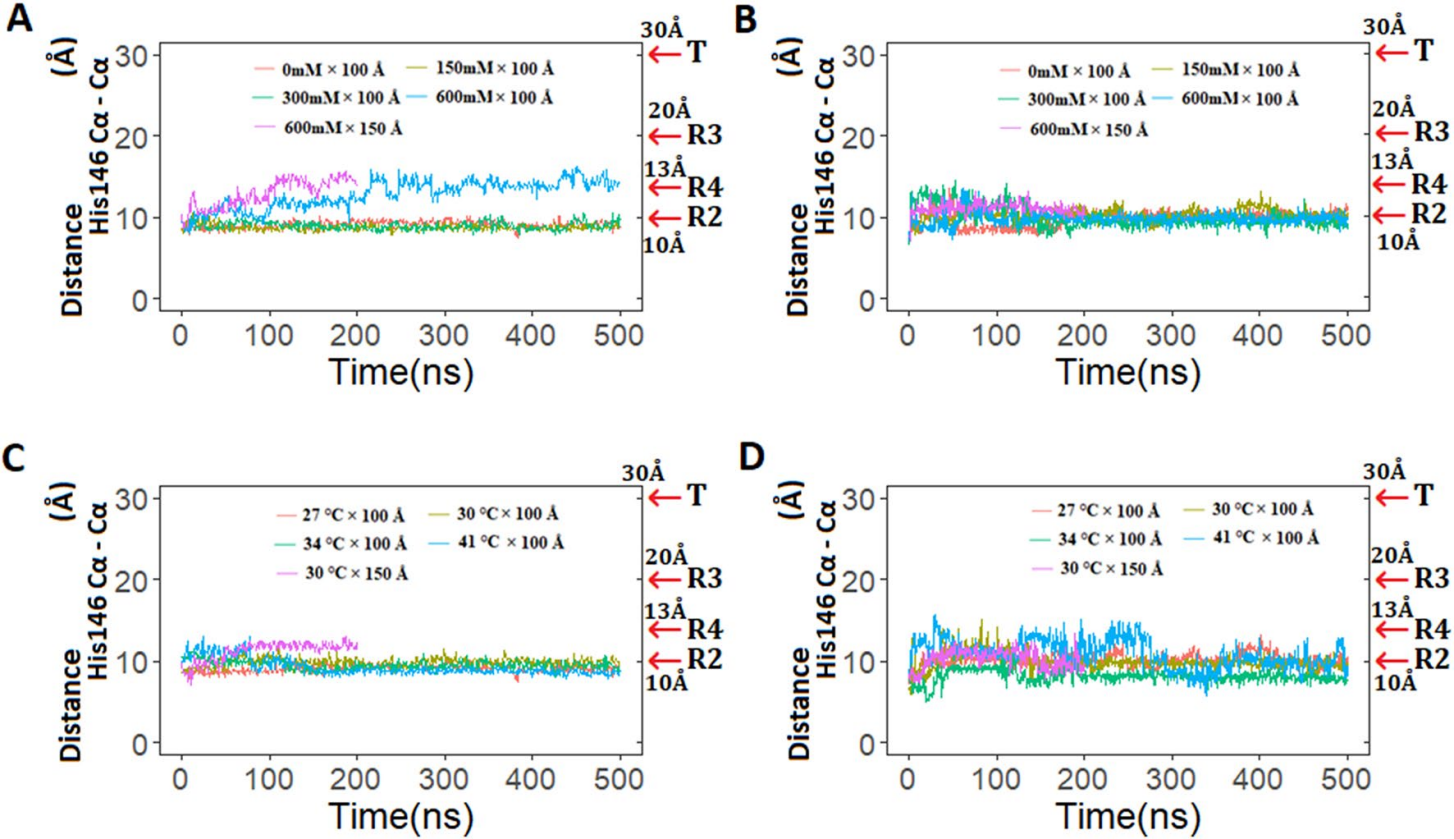
The effect of different simulated conditions on structural changes. (**A**) Distance between Cα atoms of β subunit terminal histidines (His146) of camel hemoglobin at different salt conditions; (**B**) distance between Cα atoms of β subunit terminal histidines (His146) of human hemoglobin at different salt conditions; (**C**) distance between Cα atoms of β subunit terminal histidines (His146) of camel hemoglobin at different temperature conditions; (**D**) distance between Cα atoms of β subunit terminal histidines (His146) of human hemoglobin at different temperature conditions. Graphs were plotted using R version 4.0.5 (https://www.r-project.org).

Very few MD simulation studies have been performed to evaluate R → T transition of human hemoglobin using standard physiological conditions for shorter period of time^23,24^. To investigate the role of different physiological conditions on R → T transition and/or stability of the R state of camel and human hemoglobin, we performed 500 ns simulations at different dehydrated conditions in a 100 Å box size. As a test case, a few simulations in 150 Å boxes were also carried out for 200 ns to determine the effect of box size on the stability of the R state. The terminal histidine residues (His146) of β subunits were doubly protonated in all simulations, which play an important role in the Perutz model^38^. The C_α_ His146_β1_–His146_β2_ distance is an important indicator to identify T → R and R → T transitions. Here, R → T transitions were not observed in any of the simulations (Fig. 2 and Supplementary Figures S5, S6, S7, S8). Since no transition was observed in a 100 Å box simulations and structures were found to be stable throughout the simulations, only selected conditions (600 mM and 30 °C), where 100 Å box size was assumed to have some effect on R state stability, were simulated in a 150 Å box for 200 ns to study the impact, if any. No significant difference was observed in simulation runs that were performed either at 100 Å and/or 150 Å box size. However, it is important to note that a potential R2 → R4 transition was observed in camel hemoglobin at very high salt concentration (600 mM). Interestingly, this trend was observed in both 100 Å and 150 Å box size simulations (Fig. 2). Overall, our results indicate that the R state of camel and human hemoglobin was stable throughout the simulation runs in both 100 Å and 150 Å boxes. Moreover, structures obtained at the end of 500 ns simulations, were extracted and superposed on the crystal structure that is more similar. The R structure (PDB ID: 1BBB) and T structure (PDB ID: 2DN2) for human Hb, and R structure (PDB ID: 3GDJ) for camel Hb, were used for the comparison (Table 2 and Supplementary Figures S5, S6, S7, S8). Results indicated that all simulated structures were close to their relaxed states.

**Table 2:** Cα RMSD (in Å) of the last frame of the MD simulation (at 500 ns) compared to 1BBB, 2DN2 and 3GDJ X-ray crystal structures.

Organism	Human		Camel
Structure	C_α_ RMSD (Å) compared to 1BBB (R)	C_α_ RMSD (Å) compared to 2DN2 (T)	C_α_ RMSD (Å) compared to 3GDJ (R)
2DN2	3.33	–	–
1BBB	–	3.33	–
1BBB—0 mM (100 Å box)	1.85	4.82	–
1BBB—150 mM (100 Å box)	2.03	4.87	–
1BBB—300 mM (100 Å box)	1.38	3.90	–
1BBB—600 mM (100 Å box)	1.54	4.06	–
1BBB—600 mM (150 Å box)	1.70	3.88	
1BBB—27 °C (100 Å box)	2.03	4.87	–
1BBB—30 °C (100 Å box)	1.94	4.66	–
1BBB—30 °C (150 Å box)	1.99	4.96	
1BBB—34 °C (100 Å box)	1.83	4.65	–
1BBB—41 °C (100 Å box)	1.88	4.64	–
3GDJ—0 mM (100 Å box)	–	–	1.66
3GDJ—150 mM (100 Å box)	–	–	1.52
3GDJ—300 mM (100 Å box)	–	–	1.60
3GDJ—600 mM (100 Å box)	–	–	1.73
3GDJ—600 mM (150 Å box)			1.69
3GDJ—27 °C (100 Å box)	–	–	1.52
3GDJ—30 °C (100 Å box)	–	–	1.64
3GDJ—30 °C (150 Å box)			1.61
3GDJ—34 °C (100 Å box)	–	–	1.49
3GDJ—41 °C (100 Å box)	–	–	1.82

### Comparison of camel and human hemoglobin at different salt concentrations

Since no major state transition was observed in 100 Å and 150 Å boxes and the structures were observed to be stable throughout the simulations in 100 Å boxes, these simulations were further extended up to 500 ns for further analysis.

**Figure 3:**
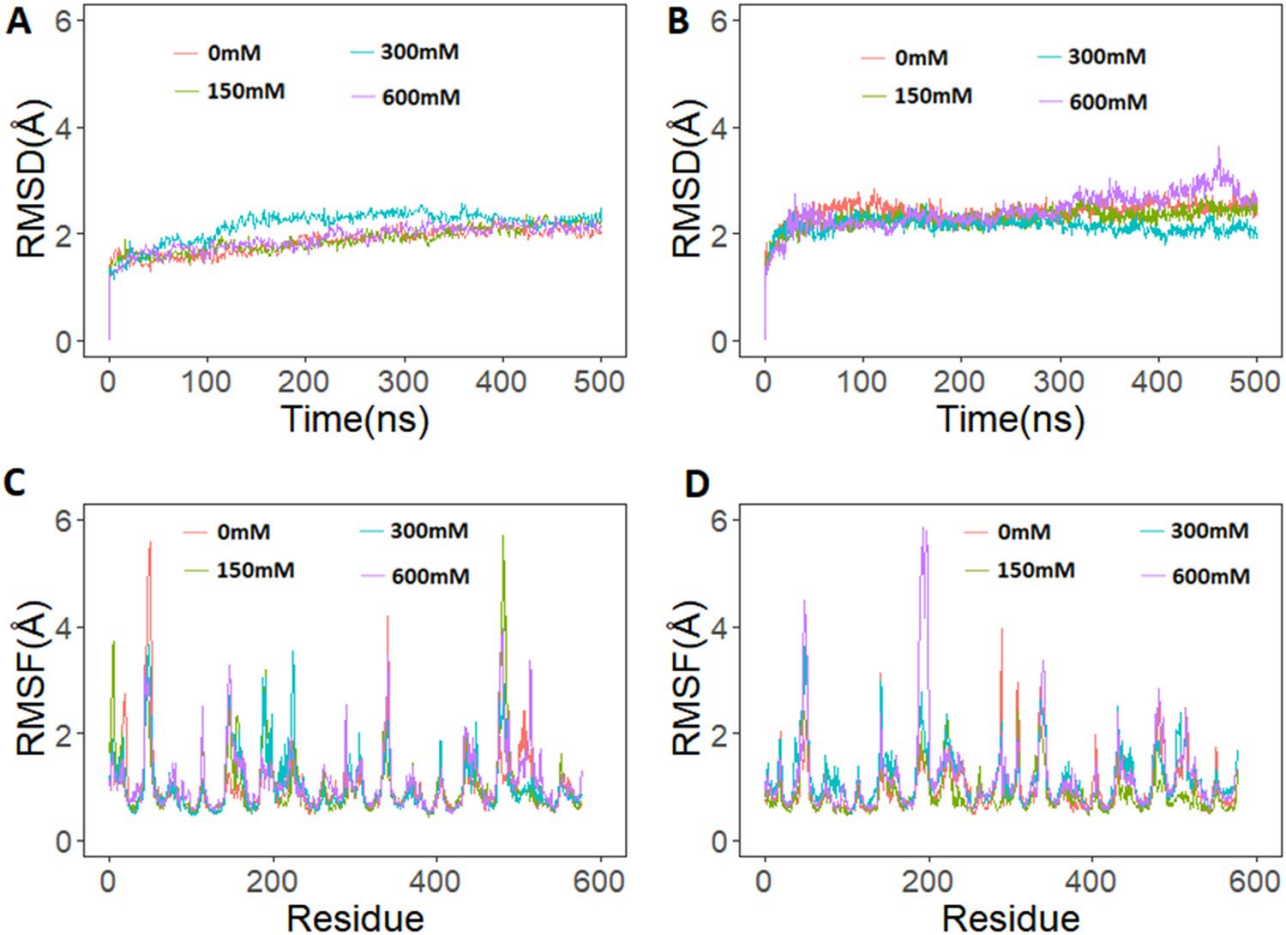
Root mean square standard deviation (RMSD) and root mean square fluctuation (RMSF) from 500 ns runs of camel and human hemoglobin simulation at different salt conditions. (**A**) RMSD of camel hemoglobin backbone atoms; (**B**) RMSD of human hemoglobin backbone atoms; (**C**) RMSF of camel hemoglobin Cα atoms and, (**D**) RMSF of human hemoglobin Cα atoms. The residue number used in the RMSF plots uses a sequential combination of α1(1–141), β1(142–287), α2 (288–428), and β2 (429–574) based on the PDB structure. Graphs were plotted using R version 4.0.5 (https://www.r-project.org).

Backbone RMSD of hemoglobin protein was computed and plotted for all systems (Fig. 3). Camel hemoglobin RMSD was under 2.0 Å for all salt concentrations except for 300 mM. All systems reached equilibrium after a few nanoseconds (Fig. 3A). The average calculated RMSD values for camel hemoglobin were 1.85 Å, 1.88 Å, 2.15 Å and 1.91 Å for the salt concentration of 0 mM, 150 mM, 300 mM and 600 mM, respectively. Notably, human hemoglobin showed higher RMSD values at all salt concentration (Fig. 3B). All systems stabilized under 2.5 Å. The average RMSD values of human hemoglobin were 2.41 Å, 2.31 Å, 2.24 Å, and 2.43 Å for the salt concentrations of 0 mM, 150 mM, 300 mM and 600 mM, respectively. Additionally, all human hemoglobin systems required slightly more time to reach equilibrium. This suggests that camel hemoglobin structure remained more stable at different salt concentrations, compared to human hemoglobin.

To evaluate and compare residue-level flexibility of camel and human hemoglobin at various salt concentrations, the RMSF of protein Cα atoms were calculated and plotted. The RMSF of the camel hemoglobin structure exhibited a similar pattern of fluctuations at different salt concentrations and high peaks were observed in loop and turn regions (Fig. 3C). The residue numbering used in RMSF plots of hemoglobin shows the sequential combination of residues from the four chains (α1, β1, α2, and β2) in the structure. Importantly, camel hemoglobin residues α1:43–53 showed more fluctuations at 0 mM salt concentration. α2:46–55 (represented by residues 333–342 in Fig. 3C) showed high fluctuations at higher salt concentrations. β1:47–53 exhibited more fluctuations at only 150 mM and 300 mM. However, the same region of β2 showed higher fluctuations at 150 mM and 600 mM. The residue His58 and His87 of α subunits and His63 and His92 of β subunits showed limited fluctuations at all salt concentrations (Fig. 3C). His87 and His92 of α and β subunits, respectively, are essential for the binding heme, while His58 and His63 of α and β subunits, respectively, play an important role in stabilizing the bound oxygen.

Human hemoglobin residues exhibited more fluctuations compared to camel hemoglobin at all salt concentrations (Fig. 3D). Loop region residues 44–52, and 70–77 of α1 subunit exhibited more fluctuations particularly at 300 mM and 600 mM. Helical residues 78–91 of α1 subunit also showed higher fluctuations at higher salt concentrations. Interestingly, the same helical residues of camel α1 subunit demonstrated extremely low fluctuations at all salt concentrations (Fig. 3C, D). However, such fluctuations were not observed in the same residues of both camel and human α2 subunits. Residues 136–141 of α1 and α2 (333–342 in Fig. 3D) of human hemoglobin exhibited higher fluctuations at lower (0 mM) and higher salt concentrations, especially at 300 mM. However, the same residues in camel hemoglobin demonstrated very limited variations at all salt concentrations except for Lys139 of α2. Residues 1–20 of β1 (142–161 in Fig. 3D) and β2 (428–448 in Fig. 2D) subunits of human hemoglobin showed limited fluctuations at different salt concentrations when compared to camel hemoglobin (Fig. 3C, D). Loop residues 45–62 (187–201 in Fig. 3C) of β1 subunit of human hemoglobin exhibited higher variations at all salt concentrations especially at 300 mM and 600 mM, compared to camel β1 subunit. However, such a trend was observed in 44–58 residues (473–486 in Fig. 3D) of β2 subunit of human hemoglobin at 300 mM. The same residues in camel hemoglobin showed more fluctuations at higher salt concentrations (Fig. 3C, D). Human hemoglobin loop residues 86–98 of β1demonstrated higher fluctuations in human hemoglobin, compared to camel hemoglobin at all salt concentrations. However, such a trend was not observed in these residues of β2 subunit. Lys82 of both β1 and β2 chains are essential for binding of 2,3-BPG and ATP^14^. These energy rich molecules interact with hemoglobin and substantially decreases its affinity for oxygen^15^. His87 and His92 are the critical residues of α and β subunits that are part of the binding site for heme. Notably, these residues fluctuated more in human hemoglobin at all salt concentrations. Residues 141–146 of β1 and β2 chains of human hemoglobin exhibited higher fluctuations particularly at higher salt concentrations. However, camel hemoglobin demonstrated limited movement at all salt concentrations (Fig. 3C, D). His143 and His146 of β1 and β2 chains are also important for the binding of 2,3-BPG and ATP^39^. Overall, human hemoglobin showed higher fluctuations at all salt concentrations, compared to camel hemoglobin.

### Comparison of camel and human hemoglobin at different temperature conditions

**Figure 4:**
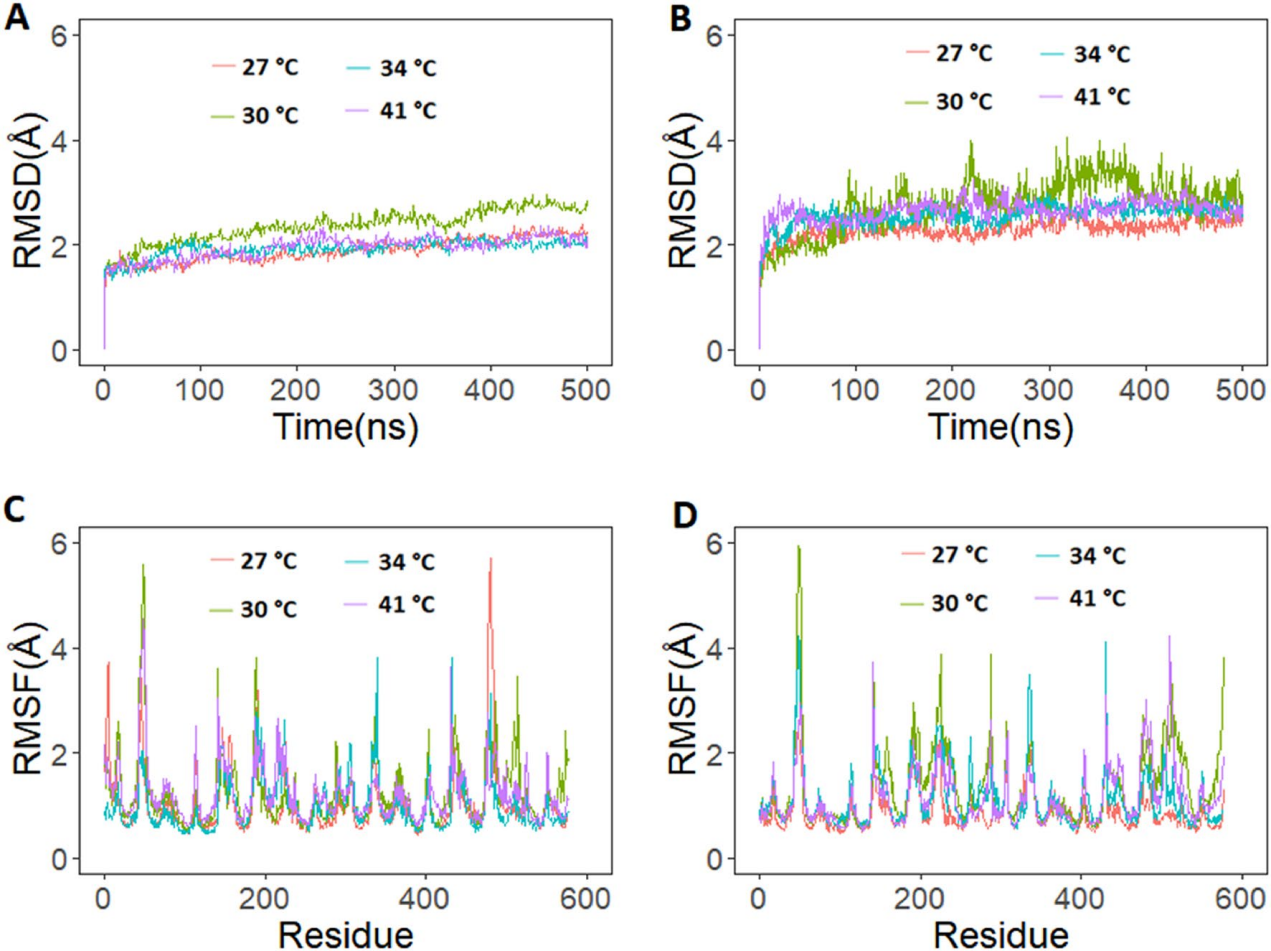
Root mean square standard deviation (RMSD) and root mean square fluctuation (RMSF) from 500 ns runs of camel and human hemoglobin simulation at different temperature conditions. (**A**) RMSD of camel hemoglobin backbone atoms; (**B**) RMSD of human hemoglobin backbone atoms; (**C**) RMSF of camel hemoglobin Cα atoms and, (**D**) RMSF of human hemoglobin Cα atoms. The residue number used in the RMSF plots uses a sequential combination of α1 (1–141), β1 (142–287), α2 (288–428), and β2 (429–574) based on the PDB structure. Graphs were plotted using R version 4.0.5 (https://www.r-project.org).

Camel and human hemoglobin proteins were simulated for 500 ns at four different temperature conditions (27 °C, 30 °C, 34 °C, and 41 °C). RMSD of backbone atoms of camel and human hemoglobin are provided in Fig. 4. RMSD of camel hemoglobin with respect to the initial structure stabilized under 2.0 Å in all the simulations except for 30 °C. Thus, camel hemoglobin structure demonstrably remained stable from the lowest to the highest temperature considered here. The average RMSD values were 1.88 Å, 2.35 Å, 1.91 Å and 1.94 Å for the temperatures 27 °C, 30 °C, 34 °C and 41 °C, respectively. Camel hemoglobin structure equilibrated within the initial few nanoseconds of all simulation (Fig. 4A). In comparison to camel hemoglobin, human hemoglobin took longer time to reach equilibrium at all temperatures considered (Fig. 4B). The RMSD stabilized under 3.0 Å in all simulations which was higher when compared to camel hemoglobin (Fig. 4A, B). The average RMSD values were 2.36 Å, 2.76 Å, 2.56 Å and 2.65 Å for the temperatures 27 °C, 30 °C, 34 °C and 41 °C, respectively.

To evaluate and compare residue-level flexibility of camel and human hemoglobin at various temperatures, the RMSF of protein Cα atoms were calculated and plotted. Camel hemoglobin structure exhibited more fluctuations at higher temperature conditions, especially residues 44–52 of α1 and α2 (332–339 in Fig. 4C) subunits, compared to other residues (Fig. 4C). The β1 helical residues 1–7 (142–149 in Fig. 4C) demonstrated higher fluctuations at higher temperature conditions, especially at 41 °C. Contrastingly, the same residues of β2 subunits showed more fluctuations at lower temperature conditions. Loop residues 44–54 of β1 (186–195 in Fig. 3C) and β2 (473–482 in Fig. 4C) showed higher fluctuations at 27 °C and 30 °C. Loop residues 72–82 (213–223 in Fig. 4C) of β1 exhibited higher fluctuations at only 34 °C and 41 °C. The same residues of β2 exhibited more fluctuations at 30 °C. The helical residues 2–7 (290–295 in Fig. 4C) and loop residues 15–19 (302–306 in Fig. 4C) of α2 showed higher fluctuations at 34 °C and 41 °C, respectively. However, the same residues of α1 subunit exhibited limited fluctuations at all temperature conditions. His87 of α subunits and His92 of β subunits fluctuated more at higher temperatures. These residues are essential for the binding of heme. Importantly, conserved residues α1:Phe43 and α1:Phe46 fluctuated more at higher temperature conditions (Fig. 4C). These functional residues are present in the heme pocket, distal to heme, where oxygen binds^40^. Additionally, conserved residues of α1—Leu86 and Leu91 also exhibited higher fluctuations at higher temperatures (Fig. 4C). These residues are part of the proximal heme pocket residues^41^.

The helical residues 1–10 and loop residues 11–17 of α1 of human hemoglobin showed slightly lower fluctuations at higher temperature conditions when compared to camel hemoglobin (Fig. 4D). The loop region residues 41–53 of α1 and α2 (328–338 in Fig. 4D) of human Hb showed lower fluctuations at different temperature conditions, compared to camel hemoglobin (Fig. 4C, D). However, the β1 subunit residues (185–194 in Fig. 4D) of human Hb exhibited higher fluctuations at higher temperature conditions, compared to camel Hb. Loop residues 44–53 of β1 of human hemoglobin exhibited lower fluctuations at lower temperature conditions, compared to camel hemoglobin. Loop residues 80–95 of human β1 (221–236 in Fig. 4D) and β2 (508–523 in Fig. 4D) exhibited higher fluctuations at higher temperature conditions compared to camel hemoglobin. Overall, in comparison to camel hemoglobin, human hemoglobin showed more fluctuations at the different temperature conditions considered here.

### Comparison of protein-heme interactions in camel and human hemoglobin

**Figure 5:**
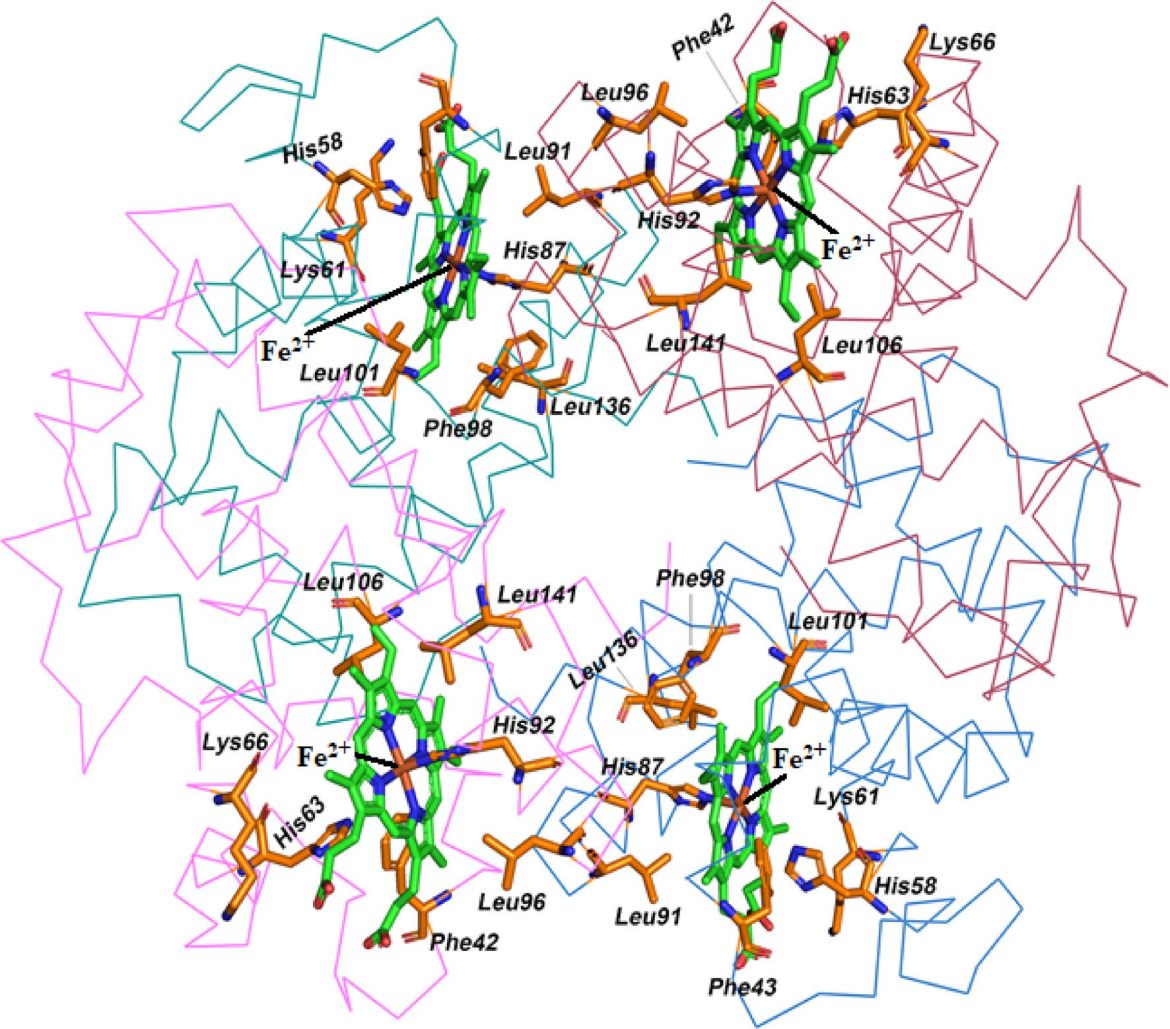
Structures of camel hemoglobin bound to heme. Protein backbone is shown in line representation and hemoglobin subunits are colored differently—α1 (blue), α2 (green), β1 (brown), and β2 (pink). Heme is shown in green stick representation and residues it interacts with are represented in orange stick representation. Image was rendered using Schrödinger Maestro 2019-4 (Schrödinger, LLC, New York, NY).

MD simulation data illustrated the formation, breakage, and reformation of several intermolecular contacts during the simulations. Some of these contacts were more stable than others. The residues of camel and human hemoglobin that formed consistent interactions with heme are shown in Fig. 5 and the duration of specific intermolecular contacts between residues of camel/human hemoglobin and heme, and the dynamics of the salient ones along the length of the simulation trajectories are provided in Supplementary Tables S2 and S3.

**Figure 6:**
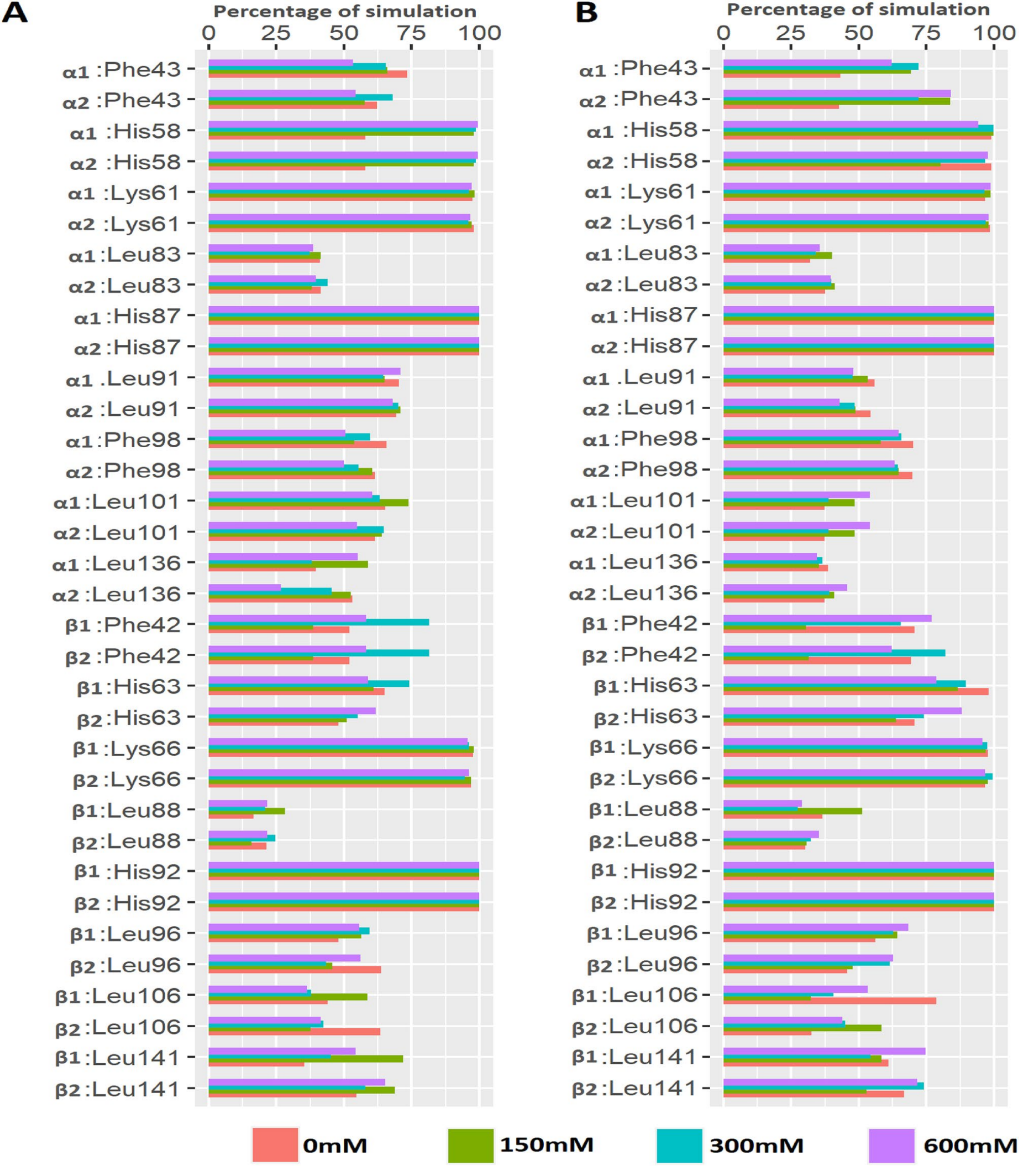
Percentage of simulation time during which human and camel hemoglobin residues maintained contact with heme. (**A**) Interaction between human hemoglobin and heme molecule at different salt concentrations. (**B**) Interaction between camel hemoglobin and heme molecule at different salt concentrations. Graphs were plotted using R version 4.0.5 (https://www.r-project.org).

**Figure 7:**
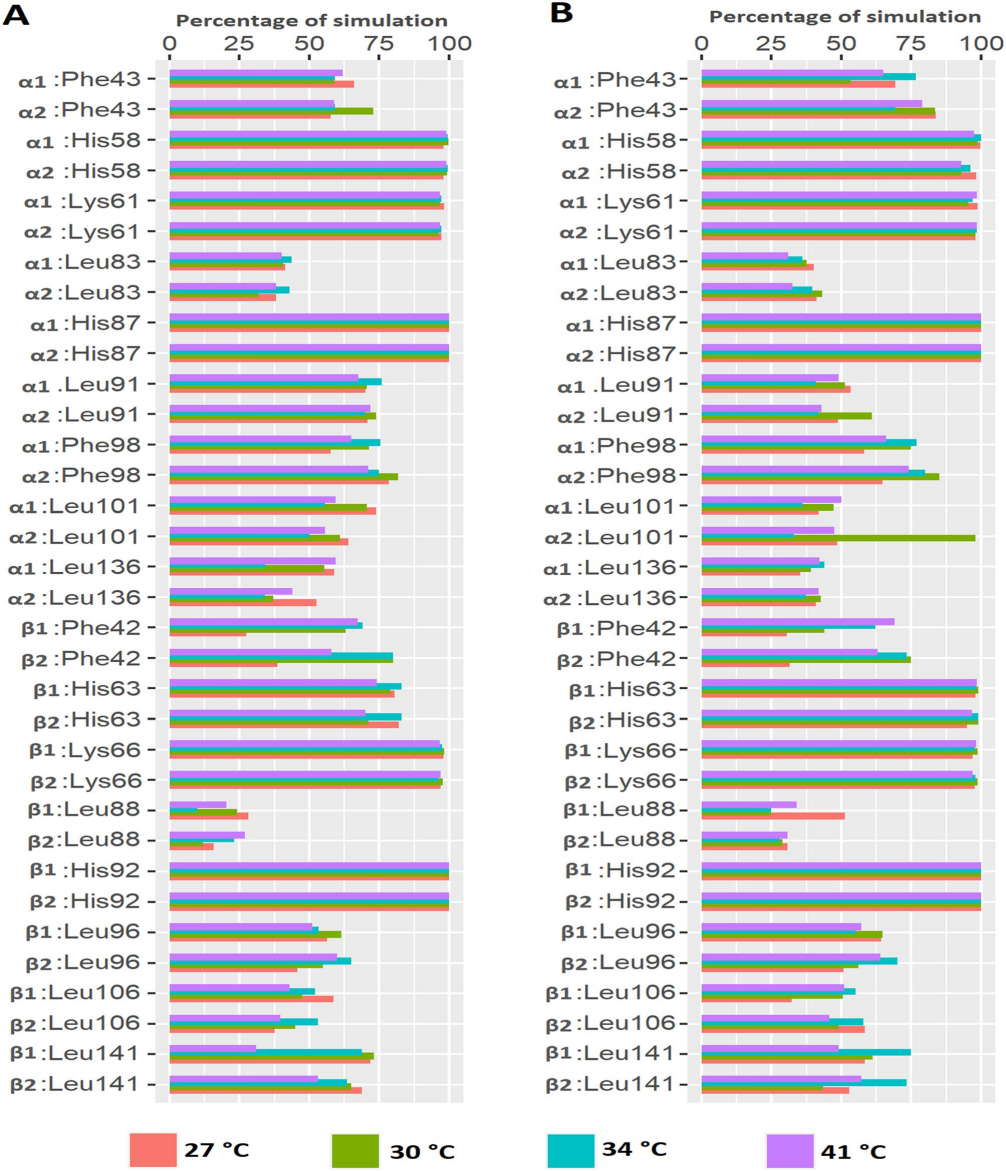
Percentage of simulation time during which human and camel hemoglobin residues maintained contact with heme. (**A**) Interaction between human hemoglobin and heme molecule at different temperatures. (**B**) Interaction between camel hemoglobin and heme molecule at different temperatures. Graphs were plotted using R version 4.0.5 (https://www.r-project.org).

His87 and His92 of hemoglobin α and β chains, respectively, interact with heme molecule. These residues consistently interacted with heme in both human and camel hemoglobin at all studied temperature and salt conditions (Figs. 6, 7). α1:Phe43 residue of human hemoglobin showed sustained interactions with heme at lower salt and temperature conditions when compared to stressed conditions. However, the equivalent residue in camel hemoglobin formed consistent interactions with heme at all studied conditions. Additionally, camel α1:Phe43 interacted more stably with heme at higher temperature conditions when compared to the human structure. During various salt concentration simulations, human α1:Phe43 showed more sustained interaction with heme, compared to camel (Fig. 6). Importantly, camel α2:Phe43 bound more stably with heme in all simulations. Such a consistent binding pattern was not observed in the equivalent residue in human simulations. The interaction of Phe43 with heme is important in anchoring the heme in its binding pocket^41^. However, it is not known if it has any significance in modulating oxygen binding to hemoglobin.

Leu91, a conserved residue of α1 chain of human and camel hemoglobin, interacted intermittently with heme in all simulations. Additionally, α1:Leu86 did not form a stable interaction with heme in both camel and human simulations. Leu86 of α1 is part of the proximal heme pocket residues and has been reported to interact with heme^40^. Instead of Leu86, Leu83 residue of α1 chain showed intermittent interaction with heme during all simulations. Apart from these residues, His58 and Lys61 of α chains in both camel and human hemoglobin interacted consistently with heme throughout the simulation at all temperature and salt conditions (Figs. 6, 7). Leu101 and Leu136 of α subunits formed comparable intermittent interactions with heme in both camel and human hemoglobin at all studied conditions. Notably, Phe98 of camel α subunits formed sustained interaction with heme in all simulations when compared to human hemoglobin (Figs. 6, 7). Phe98 is important for anchoring heme inside its binding pocket^41^.

Among other interacting residues of β chains, Leu106 of camel hemoglobin bound stably with heme in all simulations when compared to the human structure. Importantly, such interactions were observed to be more sustained at lower salt and temperature conditions when compared to higher dehydrated conditions (Figs. 6, 7). Leu141 formed a consistent interaction with heme in all simulation runs. However, such an interaction was observed to be more stable in camel hemoglobin, especially at higher temperature and salt conditions when compared to human. Leu96 of β1 and β2 chains exhibited different binding behavior with heme. This interaction was found to be more sustained in camel hemoglobin, particularly with its β1 chain at different salt and temperature conditions when compared to human hemoglobin. Lys66 residue of β subunits consistently interacted with heme in all simulations. Heme exhibited different binding behavior with Phe42 residue of β chains. This interaction, especially with β1 chain, was stable in camel, particularly at higher salt and temperature conditions, when compared to human (Figs. 6, 7).

To look at the energetics of binding, ΔG_bind_ for heme binding to human and camel hemoglobin was calculated using the MM-GBSA method using frames extracted every 100 ns from MD simulations. The heme bound to α1 subunits of camel hemoglobin showed slightly better ΔG_bind_ at higher salt conditions when compared to human hemoglobin, while heme bound to α2 subunits of human hemoglobin exhibited slightly better ΔG_bind_ at all salt concentrations. However, heme bound β subunits of camel hemoglobin showed better ΔG_bind_ at all salt conditions but 0 mM when compared to human hemoglobin (Table 3). At different temperature conditions, both α and β bound subunits of camel hemoglobin demonstrated better ΔG_bind_ in all simulations (Table 4).

**Table 3:** Estimated MM-GBSA based binding free energy (in kcal/mol) of heme binding to camel and human hemoglobin at different salt conditions.

Hemoglobin subunit	0 mM		150 mM		300 mM		600 mM	
	Camel	Human	Camel	Human	Camel	Human	Camel	Human
α1	− 126.76 ± 9.86	− 129.32 ± 5.30	− 130.37 ± 8.94	− 132.34 ± 4.38	− 131.21 ± 8.06	− 128.71 ± 3.47	− 137.22 ± 8.79	− 130.55 ± 7.60
α2	− 132.46 ± 7.27	− 134.85 ± 7.84	− 128.77 ± 4.21	− 130.94 ± 5.89	− 126.45 ± 5.48	− 133.11 ± 6.12	− 131.25 ± 5.42	− 131.27 ± 6.02
β1	− 133.96 ± 6.53	− 129.68 ± 8.52	− 133.73 ± 6.91	− 124.26 ± 12.04	− 125.17 ± 3.49	− 124.58 ± 7.87	− 135.18 ± 9.23	− 127.13 ± 10.01
β2	− 119.44 ± 10.01	− 128.52 ± 4.55	− 128.25 ± 9.35	− 122.34 ± 3.78	− 133.93 ± 3.52	− 129.66 ± 7.27	− 134.56 ± 5.22	− 126.77 ± 6.00

**Table 4:** Estimated MM-GBSA based binding free energy (in kcal/mol) of heme binding to camel and human hemoglobin at different temperature conditions.

Hemoglobin subunit	27 °C		30 °C		34 °C		41 °C	
	Camel	Human	Camel	Human	Camel	Human	Camel	Human
α1	− 130.37 ± 8.94	− 132.34 ± 4.38	− 132.58 ± 8.06	− 129.35 ± 3.03	− 131.59 ± 5.76	− 128.56 ± 7.56	− 131.24 ± 8.63	− 128.04 ± 4.03
α2	− 128.77 ± 4.21	− 130.94 ± 5.89	− 133.83 ± 8.53	− 129.88 ± 6.80	− 129.92 ± 8.84	− 125.61 ± 6.80	− 132.33 ± 10.51	− 128.08 ± 1.28
β1	− 133.73 ± 6.91	− 124.26 ± 12.04	− 129.16 ± 6.57	− 125.01 ± 2.51	− 127.81 ± 3.98	− 123.16 ± 8.95	− 124.86 ± 1.83	− 118.72 ± 14.09
β2	− 128.25 ± 9.35	− 122.34 ± 3.78	− 128.67 ± 4.70	− 127.71 ± 6.47	− 131.21 ± 9.40	− 124.97 ± 12.01	− 128.57 ± 7.81	− 123.81 ± 7.94

## Discussion

This study provides insights into the stability of camel and human hemoglobin under various temperature and salt conditions, along with its interactions with heme. Several long 500 ns MD simulations were performed to assess this. No R → T transition was observed at studied conditions but camel hemoglobin exhibited a possible R2 → R4-like transition at 600 mM only. Overall, these simulations, at normal and stressed conditions, indicated that camel hemoglobin structures remained more stable when compared to human hemoglobin. Additionally, in camel hemoglobin, critical regions important for the binding of heme, and energy rich molecules such as 2,3-BPG and ATP demonstrated limited fluctuations in the conditions studied.

Generally, mammalian erythrocytes are highly sensitive to osmotic fragility with the notable exception of camels^9,18,42^. Camel RBCs are extremely resistant to osmotic fragility and are inherently adapted to endure sudden fluctuations in blood osmolarity without affecting function^43^. In order to measure the changes resulting from different salt concentrations, MD simulations were performed, since no prior attempt has been made to look at the residue level fluctuations of the camel protein. Indeed, the simulations reflect fluctuations being affected by different salt concentrations (Fig. 2C, D). Protein function is dependent on the fluctuations of the macromolecule^44,45^. Here, simulations, under different salt concentrations, demonstrated fluctuations of important regions essential for oxygen binding (Fig. 2). Interestingly, higher fluctuations were observed in residues near the heme binding sites in each subunit of human hemoglobin at higher temperature and salt conditions, compared to camel hemoglobin. Importantly, His87 of α subunits and His92 of β subunits demonstrated more fluctuations in human hemoglobin at higher salt concentrations when compared to camel hemoglobin. These residues are essential for the binding of heme groups that bind oxygen. Additionally, conserved residues α1:Phe43 and α1:Phe46 showed higher fluctuations in human hemoglobin at higher salt conditions (Fig. 2). These residues are present in the heme pocket distal to heme where oxygen binds^40^. Similarly, conserved α1 residues Leu86 and Leu91 also exhibited higher fluctuations in human hemoglobin when salt concentration was increased. These residues are part of the proximal heme pocket residues^40^. Interestingly, in comparison to human hemoglobin, camel hemoglobin demonstrated lower fluctuations at all salt concentrations. Similarly, when different temperature conditions were used camel hemoglobin showed lower fluctuations at higher temperatures especially in the regions important for the binding of heme when compared to human hemoglobin (Fig. 3C, D). Therefore, these observations may support the stability and function of camel hemoglobin under stress.

Studies have indicated the importance of the distal histidine residues α:His58 and β:His63 in the binding of heme. They also play a key role in regulating the rate and affinity of oxygen binding to human hemoglobin^3^. These residues axially coordinate heme iron and such interactions are essential for stabilizing the oxygen molecule bound to the heme iron^2,46^. Here, β:His63 of camel hemoglobin bound more stably with heme group in all simulations when compared to human hemoglobin (Figs. 5 and 6). This suggests that such sustained interactions could likely help camel hemoglobin to stably bind oxygen unlike human hemoglobin.

The dynamics of non-essential residues for the binding of heme in both human and camel hemoglobin were also evaluated. Of note, α:Phe43, α:Phe98, β:Phe42, β:Leu96, β:Leu106, and β:Leu141 residues formed sustained interactions with heme group in camel hemoglobin especially at higher salt and temperature conditions when compared to human hemoglobin (Figs. 5 and 6). α subunit residues Lys61 and Leu101 demonstrated similar binding behavior with heme in all simulations. These are important residues that line the heme binding pocket^5,20,47^. Thus, it is perceivable that these residues along with α:His58 and β:His63 could play a role in cavity structure and dynamics that determine the binding kinetics of ligands.

Moreover, heme showed higher binding affinity with camel hemoglobin at stressed conditions when compared to human hemoglobin (Tables 2, 3). This indicates the formation of stronger interactions in camel hemoglobin as indicated by ΔG_bind_ computed using the MM-GBSA method.

Interestingly, a camel’s body temperature can vary between 34 and 41 °C during the day^42,43^. Moreover, camels have the ability to live without drinking water for longer period of time. These factors in combination produces a severely dehydrated state in camels. The findings of this study are of significant importance in understanding the behavior of camel hemoglobin under different dehydrated conditions, particularly the interactions with heme during dehydrated conditions. Here, camel hemoglobin exhibited more stability at stressed conditions, compared to human hemoglobin.

## Conclusion

While the residues of camel and human hemoglobin that interact with heme are conserved and share a similar mode of interaction with heme, there are several disparities in the dynamics and energetics of these interactions. One notable difference is the formation of a more stable interaction between the distal histidine of β:His63 of camel hemoglobin and heme as well as the interaction between α:Phe43 and α:Phe98 of camel hemoglobin with heme. These were observed to be less stable in human hemoglobin simulations. However, further studies are needed to confirm the role of these interactions in tense state (T) to relaxed state (R) transition in camel hemoglobin. Binding of energy rich molecules such as 2,3-BPG and ATP are essential for the unloading of oxygen from hemoglobin. It would be interesting to perform a comparative study under stressed conditions to measure the stability and energetics of the binding of these molecules to camel and human hemoglobin.

## Supplementary Information

Supplementary Information.

## Data Availability

The datasets generated during and/or analysed during the current study are available from the corresponding author on reasonable request.
