# Supplementary material for: Dynamics of camel and human hemoglobin revealed by molecular simulations
Source: Sci Rep. 2022 Jan 7;12:122. doi: 10.1038/s41598-021-04112-y (PMC8741986; doi:10.1038/s41598-021-04112-y)
Supplement: Supplementary file 1 — Supplementary Information. [file 41598_2021_4112_MOESM1_ESM.pdf]

Dynamics of camel and human hemoglobin revealed by molecular simulations

Amanat Ali[1], Soja Saghar Soman[2] and Ranjit Vijayan [1,3,4,*]

[1] Department of Biology, College of Science, United Arab Emirates University, PO Box 15551, Al Ain, United Arab Emirates.
[2] New York University Abu Dhabi, PO Box 129188, Abu Dhabi, United Arab Emirates.
[3] The Big Data Analytics Center, United Arab Emirates University, PO Box 15551, Al Ain, United Arab Emirates.
[4] Zayed Center for Health Sciences, United Arab Emirates University, PO Box 15551, Al Ain, United Arab Emirates.

* Correspondence: Ranjit.v@uaeu.ac.ae; Tel.: +971 3 71363

Supplementary Information

Table 1. Protonation states of histidine residues of human and camel hemoglobin used during simulations.

Human hemoglobin (1BBB)				Camel hemoglobin (3GDJ)			
**His (α)**	**Protonation**	**His (β)**	**Protonation**	**His (α)**	**Protonation**	**His (β)**	**Protonation**
20	HIE	2	HID	20	HIE	2	HID
45	HIE	63	HIE	45	HIE	63	HIE
50	HID	77	HID	50	HID	77	HID
58	HIE	92	HID*	58	HIE	92	HID*
72	HID	97	HID	72	HID	97	HID
87	HID*	116	HIE	87	HID*	117	HID
89	HID	117	HID	89	HID	143	HID
103	HIE	143	HID	103	HIE	146	HIP
112	HID	146	HIP	112	HID		
122	HIE			122	HIE		

HIE (neutral, ε-nitrogen protonated), HID (neutral, δ-nitrogen protonated), HIP (+1 charged, both δ- and ε-nitrogens protonated), * Histidine bound to heme groups.

Table 2. Percentage of simulation time specific interactions were formed between heme and camel/human hemoglobin at different salt conditions.

Contact time (%)								
**Residue**	**0 mM**		**150 mM**		**300 mM**		**600 mM**	
	**Camel (3GDJ)**	**Human (1BBB)**	**Camel (3GDJ)**	**Human (1BBB)**	**Camel (3GDJ)**	**Human (1BBB)**	**Camel (3GDJ)**	**Human (1BBB)**
α1-Phe43	43.2	73.3	69.4	65.9	72.2	65.5	62.1	53.4
α2-Phe43	42.6	62.3	83.8	57.7	72	68.1	84	54.2
α1-His58	99	58	99.8	98	99.8	98.8	94.1	99.5
α2-His58	99	58	99.8	98	80.2	93	96.8	98
α1-Lys61	96.8	97.4	98.8	98.3	96.5	96.3	98.8	97.2
α2-Lys61	98.4	981	98	97.2	97	96	98	96.8
α1-Leu83	32	41.1	40.1	41.5	34	37.3	35.6	38.6
α2-Leu83	37.6	41.5	41	38	39.9	44	39.6	39.4
α1-His87	100	100	100	100	100	100	100	100
α2-His87	100	100	100	100	100	100	100	100
α1-Leu91	55.8	70.2	53.2	65.1	47.8	64.4	48	70.9
α2-Leu91	48.8	69.2	48.4	70.7	48.8	70.1	42.8	68.1
α1-Phe98	70.1	65.7	58.2	53.7	65.8	59.7	64.8	50.1
α2-Phe98	69.9	61.4	64.6	60.5	64.4	55.3	63.3	49.9
α1-Leu101	37.2	65.3	48.6	73.8	38.8	63.1	54	60.5
α2-Leu101	37.2	61.3	48.4	64	39	64.8	52	54.9
α1-Leu136	38.6	39.5	35.4	58.8	36.6	38.1	34.5	55
α2-Leu136	37.2	53.1	40.8	52.6	39.2	45.5	45.8	26.6
β1-Phe42	70.6	52.1	30.4	38.7	65.6	81.4	76.8	58.2
β2-Phe42	69.2	51.1	31.5	35.7	81.9	80.2	62.2	56.1
β1-His63	97.9	65	86.6	61	89.6	74	78.6	59
β2-His63	70.5	48	63.6	49.8	74	55	88.4	61.7
β1-Lys66	97.8	97.8	97	97.9	97.4	96.2	95.6	95.6
β2-Lys66	96.8	97	97.8	97	99.4	94.7	96.6	96.2
β1-Leu88	36.6	16.5	51.2	28.1	27.5	20.8	28.9	21.5
β 2-Leu88	30.1	21.4	30.8	15.8	32.2	24.6	35.2	21.6
β1-His92	100	100	100	100	100	100	100	100
β2-His92	100	100	100	100	100	100	100	100
β1-Leu96	56	56.4	64.3	59.5	62.8	59.5	68.3	55.5
β2-Leu96	45.6	63.8	47.8	45.8	61.5	43.4	62.6	56.1
β1-Leu106	78.8	43.8	32.2	58.7	40.5	37.7	53.6	36.3
β2-Leu106	32.6	63.5	58.5	37.5	45	42.3	44	41.3
β1-Leu141	60.9	35.2	58.3	71.9	54.4	45.2	74.6	54.2
β2-Leu141	66.7	54.5	52.8	68.9	74	58	71.6	65.3

Table 3. Percentage of simulation time specific interactions were formed between heme and camel/human hemoglobin at different temperature conditions.

Contact time (%)								
**Residue**	**27 °C**		**30 °C**		**34 °C**		**41 °C**	
	**Camel (3GDJ)**	**Human (2HHB)**	**Camel (3GDJ)**	**Human (2HHB)**	**Camel (3GDJ)**	**Human (2HHB)**	**Camel (3GDJ)**	**Human (2HHB)**
α1-Phe43	69.4	65.9	53.2	58.8	76.6	59.2	65	61.9
α2-Phe43	83.8	57.7	83.6	72.8	69.3	59.2	79	58.8
α1-His58	99.8	98	98.8	99.8	100	99.6	97.4	99
α2-His58	98.2	98	92.8	99.3	96.3	99.6	93	99
α1-Lys61	98.8	98.3	95.4	96.6	97	97.3	98.4	96.7
α2-Lys61	98	97.2	98	96.3	98.6	97.3	98.4	96.6
α1-Leu83	40.1	41.5	37.6	40.5	36	43.6	31	40
α2-Leu83	41	38	43.2	32.1	39.7	43	32.6	38
α1-His87	100	100	100	100	100	100	100	100
α2-His87	100	100	100	100	100	100	100	100
α1-Leu91	53.2	70.1	51.4	70.6	40.8	75.8	49	67.4
α2-Leu91	48.8	70.7	61	73.9	42	70	42.8	71.8
α1-Phe98	58.2	57.7	75	71.3	77	75.3	66	65
α2-Phe98	64.6	78.5	85	81.7	80	75	74	71
α1-Leu101	42	73.8	47.2	70.6	36	55.7	50	59.5
α2-Leu101	48.6	64	98	61	98	50	33	55.7
α1-Leu136	35.4	58.8	39	55.3	43.8	34.3	42.2	59.5
α2-Leu136	40.8	52.6	42.6	37	37.3	34	42	44
β1-Phe42	30.4	27.4	43.8	63	62.2	69	69	67.3
β2-Phe42	31.5	38.7	75	80	73.4	80	63	58
β1-His63	98	80.5	99	79	98.2	83	98.6	74
β2-His63	95	82	99	71	99	83	96.8	70.1
β1-Lys66	97	97.9	98.8	98.2	97.6	97.5	98.3	96.7
β2-Lys66	97.8	97	98.8	97.7	98	96.2	97	97
β1-Leu88	51.2	28.1	24.8	24	25	10	34	20.2
β2-Leu88	30.8	15.8	29	11.9	28	23	30.6	27
β1-His92	100	100	100	100	100	100	100	100
β2-His92	100	100	100	100	100	100	100	100
β1-Leu96	64.3	56.4	64.8	61.3	55	53.4	57	51
β2-Leu96	50.8	45.8	56.2	54.8	70	65	64	60
β1-Leu106	32.2	58.7	50.4	47.4	55	52	51	43
β2-Leu106	58.5	37.5	49.1	44.9	58	53	45.8	39.7
β1-Leu141	58.3	71.9	61.2	73.2	75	68.9	49	31
β2-Leu141	52.8	68.9	43.4	65.1	73.4	63.5	57.2	53

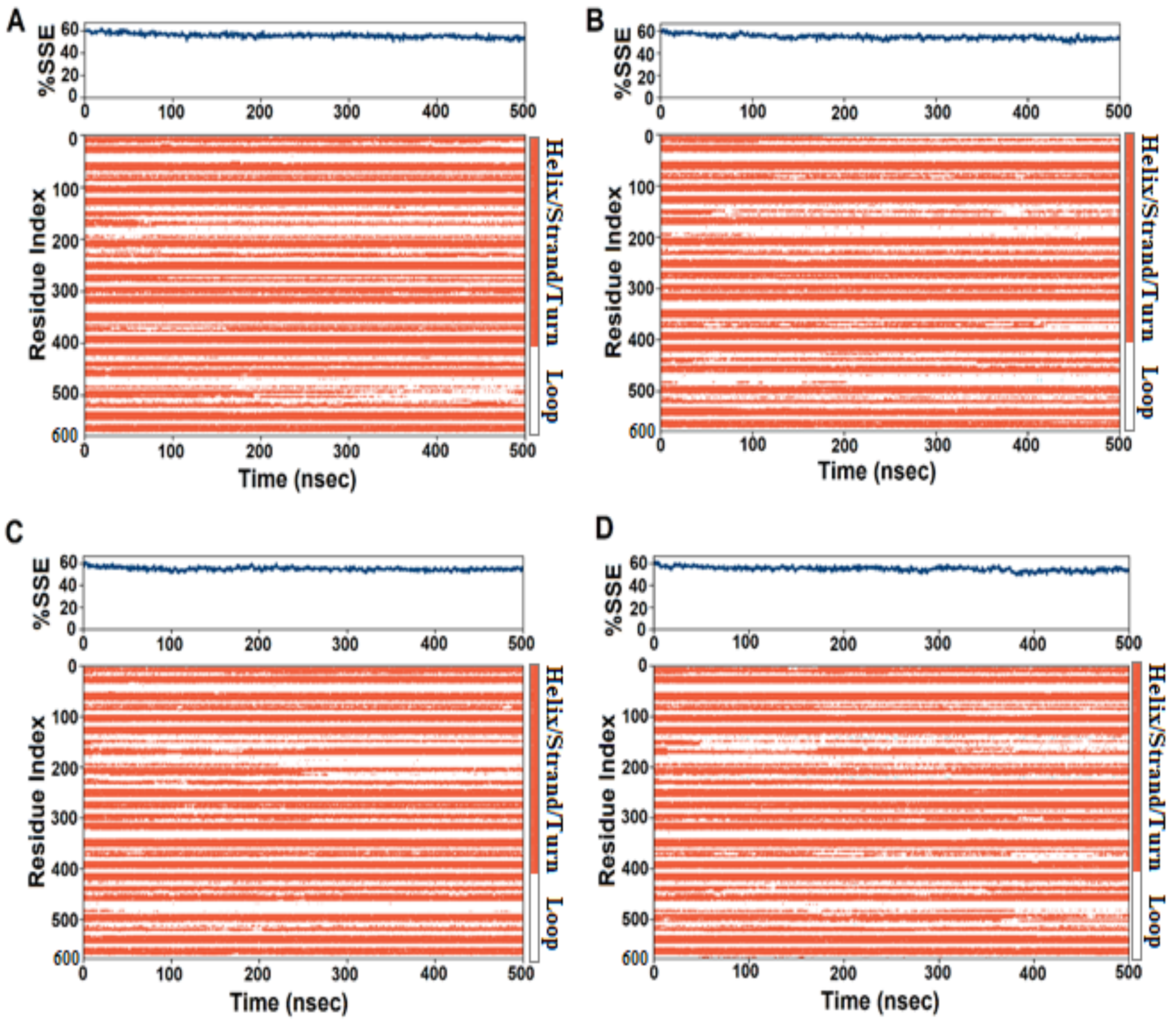

Figure 1. Secondary structure evolution of camel hemoglobin at different salt conditions. A) 0mM; B) 150mM; C) 300mM; D) 600 mM. Secondary structure elements (SSE) represented predominately by α-helices are shown in red color while loop regions are shown in white color. Images were generated using Maestro-Desmond Interoperability Tools 2019-4 (Schrödinger, LLC, New York, NY).

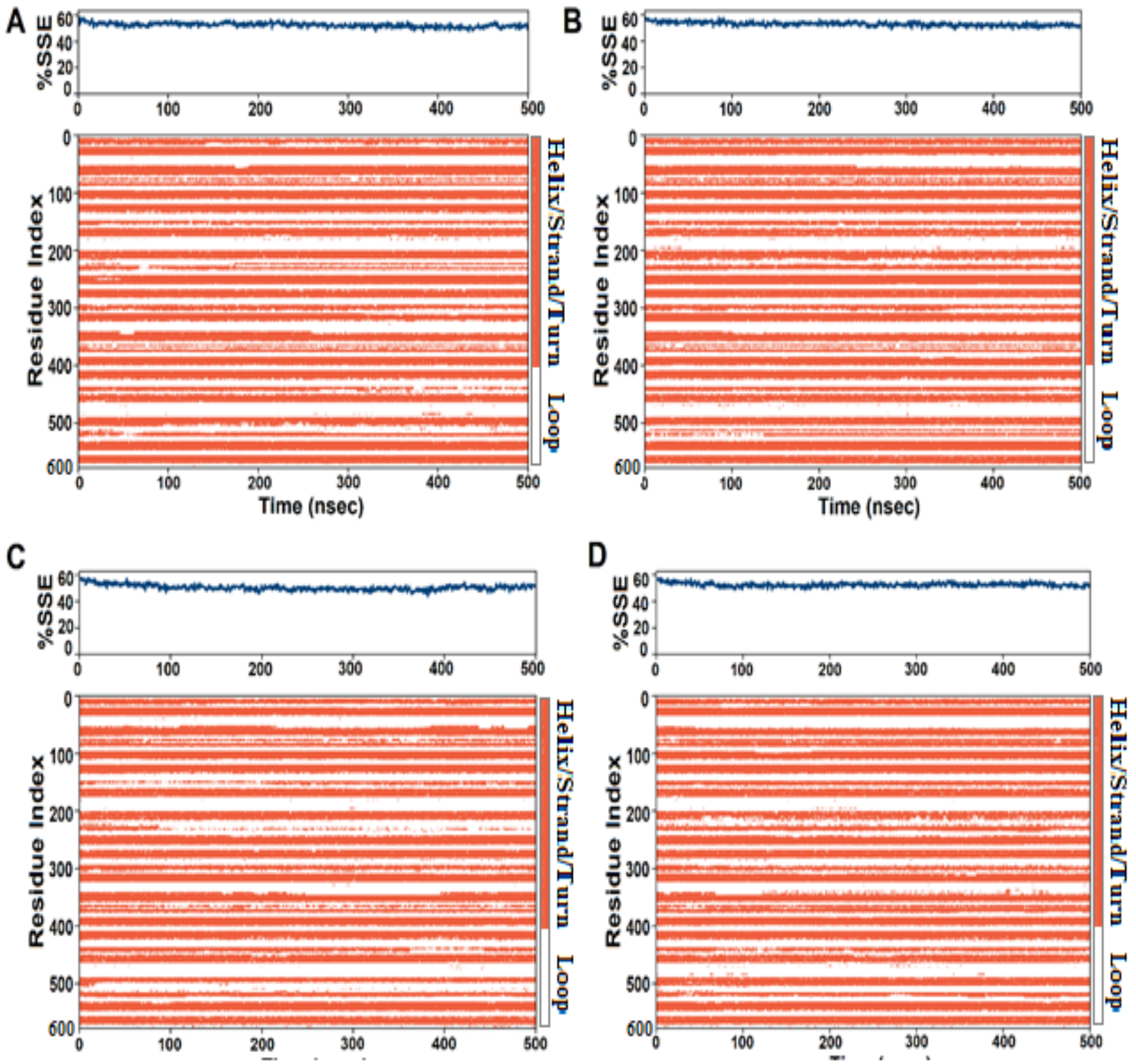

Figure 2. Secondary structure evolution of human hemoglobin at different salt conditions. A) 0mM; B) 150mM; C) 300mM; D) 600 mM. Secondary structure elements (SSE) represented predominately by α-helices are shown in red color while loop regions are shown in white color. Images were generated using Maestro-Desmond Interoperability Tools 2019-4 (Schrödinger, LLC, New York, NY).

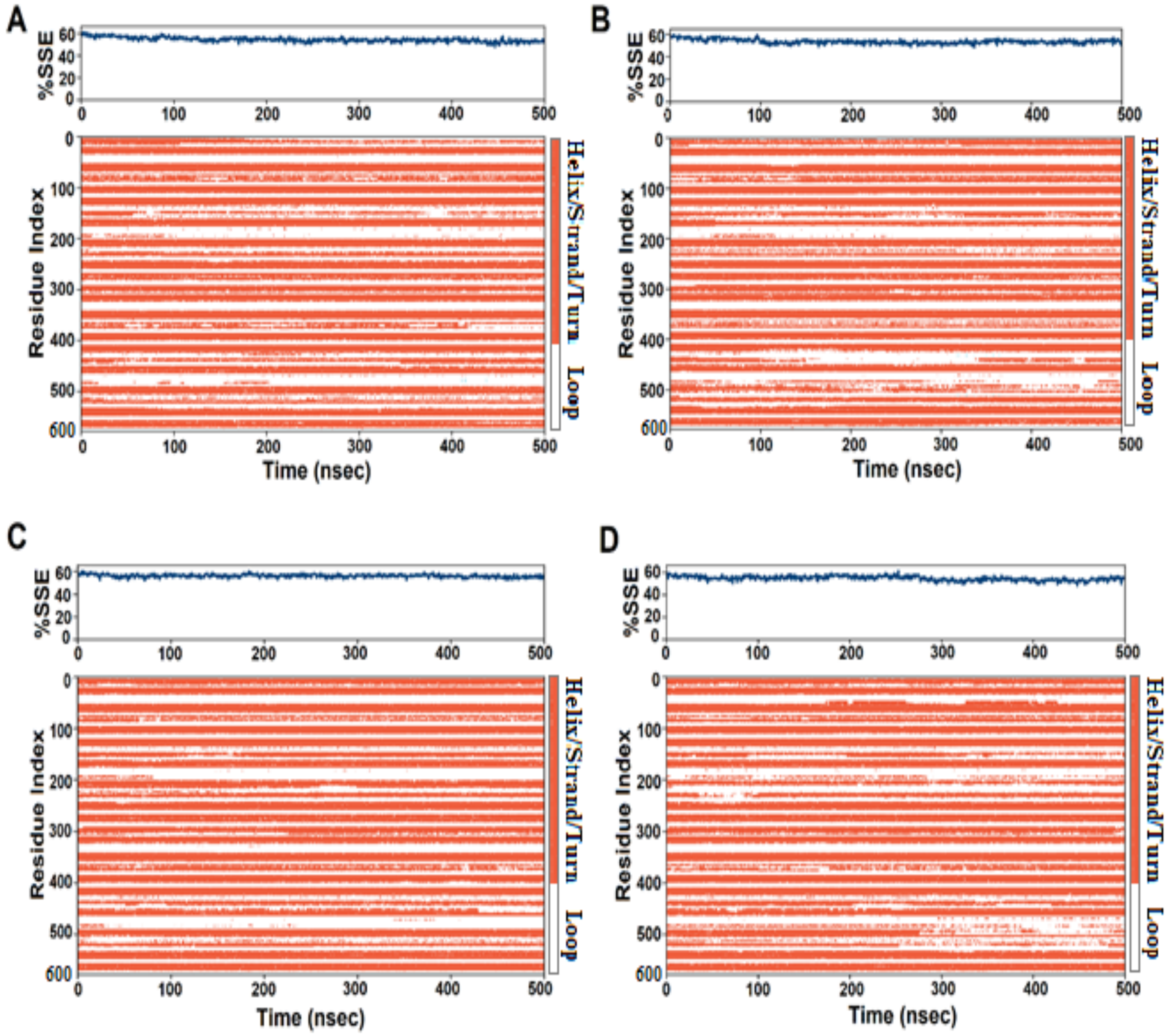

Figure 3. Secondary structure evolution of camel hemoglobin at different temperature conditions. A) 27 °C; B) 30 °C; C) 34 °C; D) 41 °C. Secondary structure elements (SSE) represented predominately by α-helices are shown in red color while loop regions are shown in white color. Images were generated using Maestro-Desmond Interoperability Tools 2019-4 (Schrödinger, LLC, New York, NY).

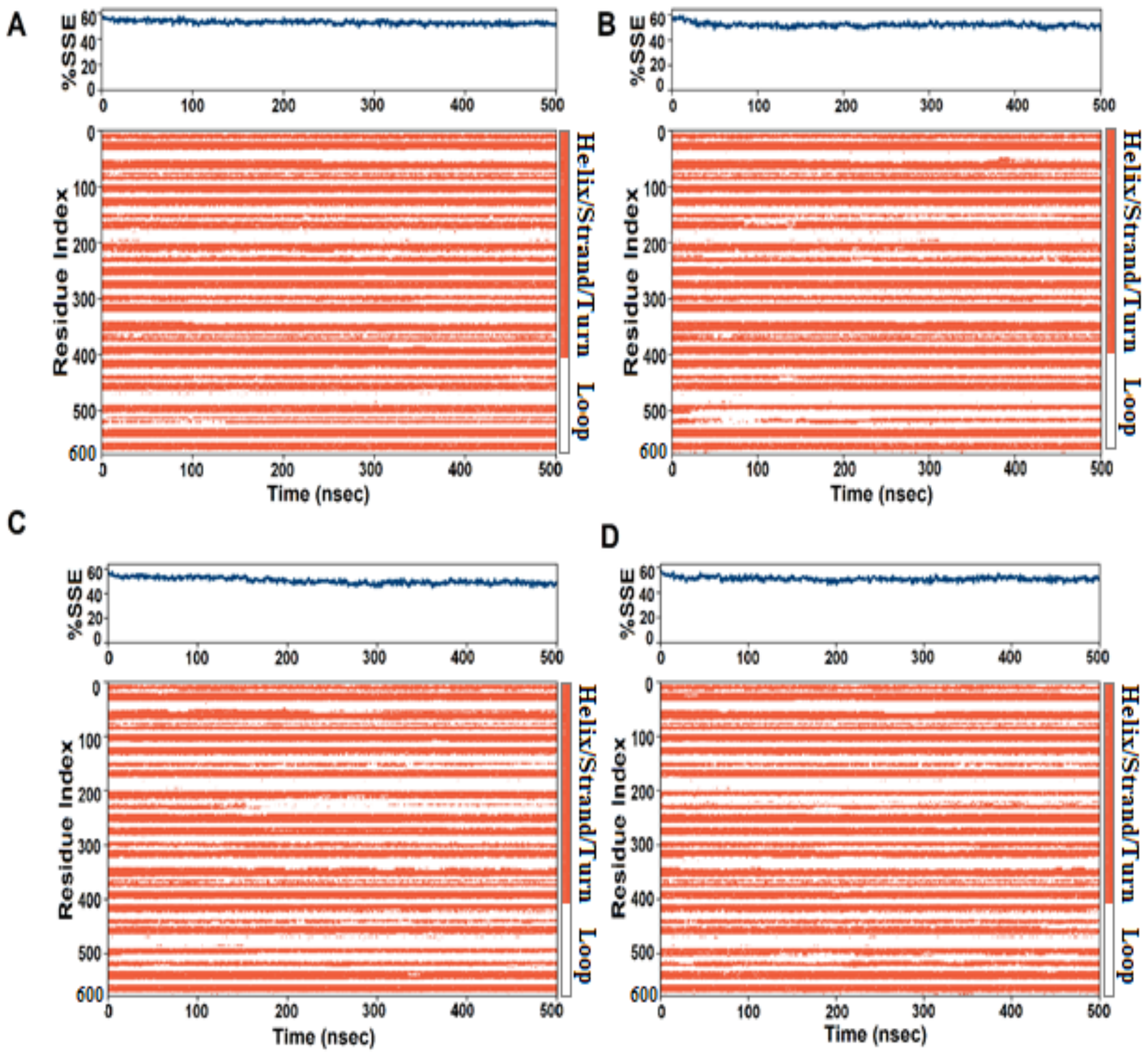

Figure 4. Secondary structure evolution of human hemoglobin at different temperature conditions. A) 27 °C; B) 30 °C; C) 34 °C; D) 41 °C. Secondary structure elements (SSE) represented predominately by α-helices are shown in red color while loop regions are shown in white color. Images were generated using Maestro-Desmond Interoperability Tools 2019-4 (Schrödinger, LLC, New York, NY).

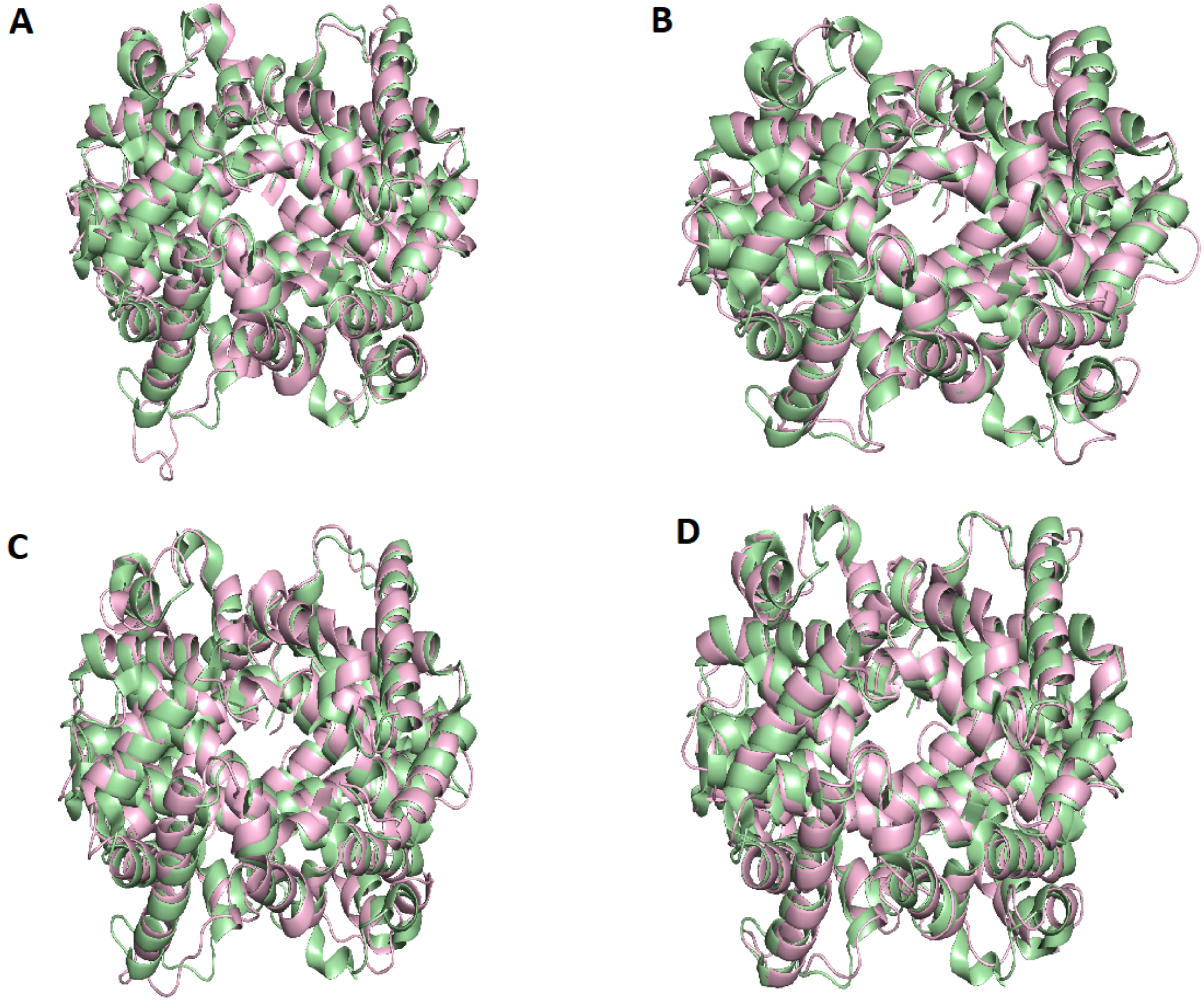

Figure 5. Effect of different salt concentrations on structure of camel Hb. Crystal structure (PDB ID: 3GDJ) and simulated structures of Hb are shown in green and pink cartoon representation, respectively. A) Superimposition of the 3GDJ structure and the Hb structure (t=500 ns) simulated at 0mM. B) Superimposition of the 3GDJ structure and the Hb structure (t=500 ns) simulated at 150mM. C) Superimposition of the 3GDJ structure and the Hb structure (t=500 ns) simulated at 300mM. D) Superimposition of the 3GDJ structure and the Hb structure (t=500 ns) simulated at 600mM. Images were generated using the PyMOL Molecular Graphics System version 2.0 (http://www.pymol.org/pymol).

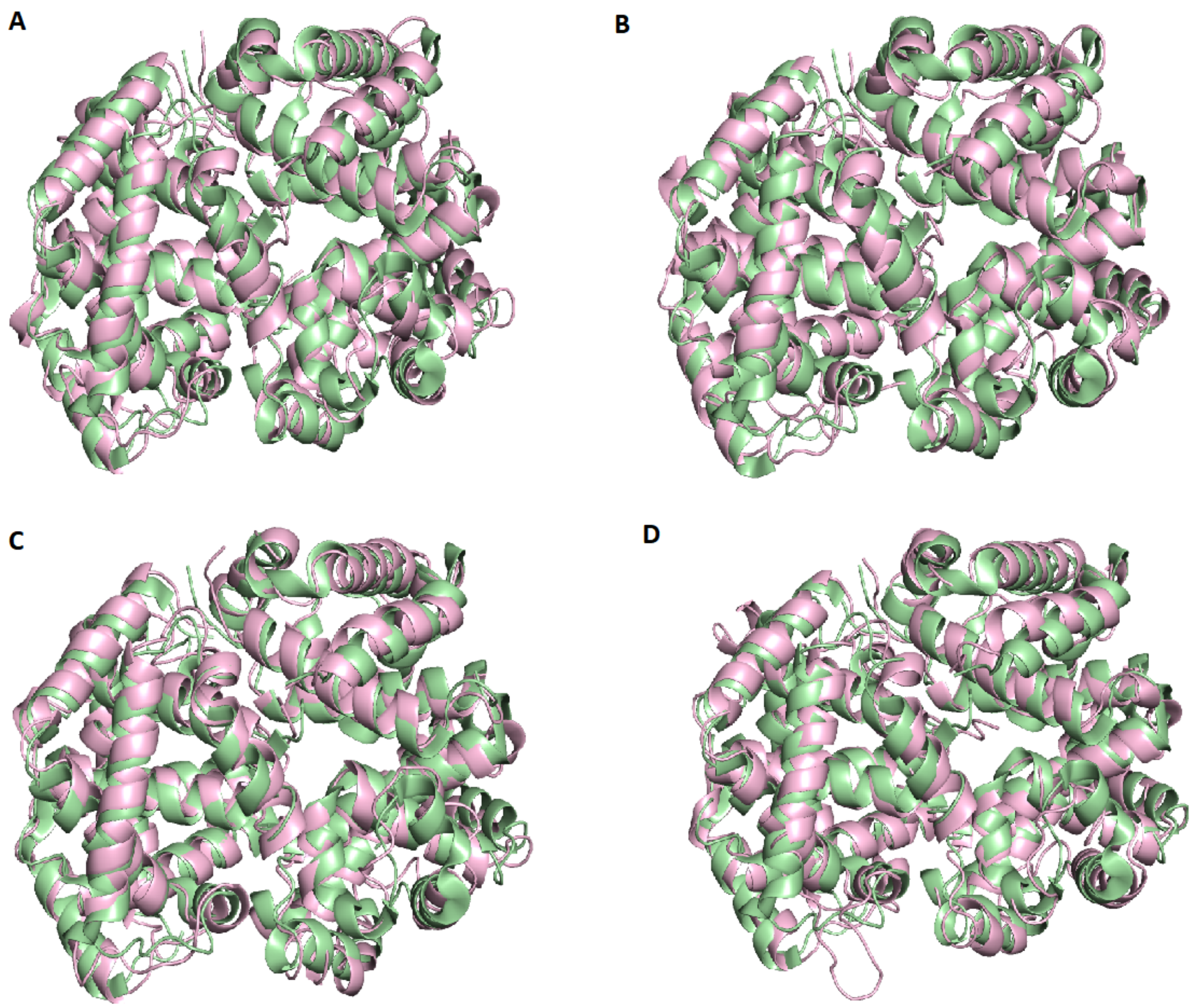

Figure 6. Effect of different salt concentrations on the structure of human Hb. Crystal struc ture (PDB ID: 1BBB) and simulated structure of Hb are shown in green and pink cartoon representation, respectively. A) Superimposition of the 1BBB structure and the Hb structure (t=500 ns) simulated at 0mM. B) Superimposition of the 1BBB structure and the Hb structure (t=500 ns) simulated at 150mM. C) Superimposition of the 1BBB structure and the Hb structure (t=500 ns) simulated at 300mM. D) Superimposition of the 1BBB structure and the Hb structure (t=500 ns) simulated at 600mM. Images were generated using the PyMOL Molecular Graphics System version 2.0 (http://www.pymol.org/pymol).

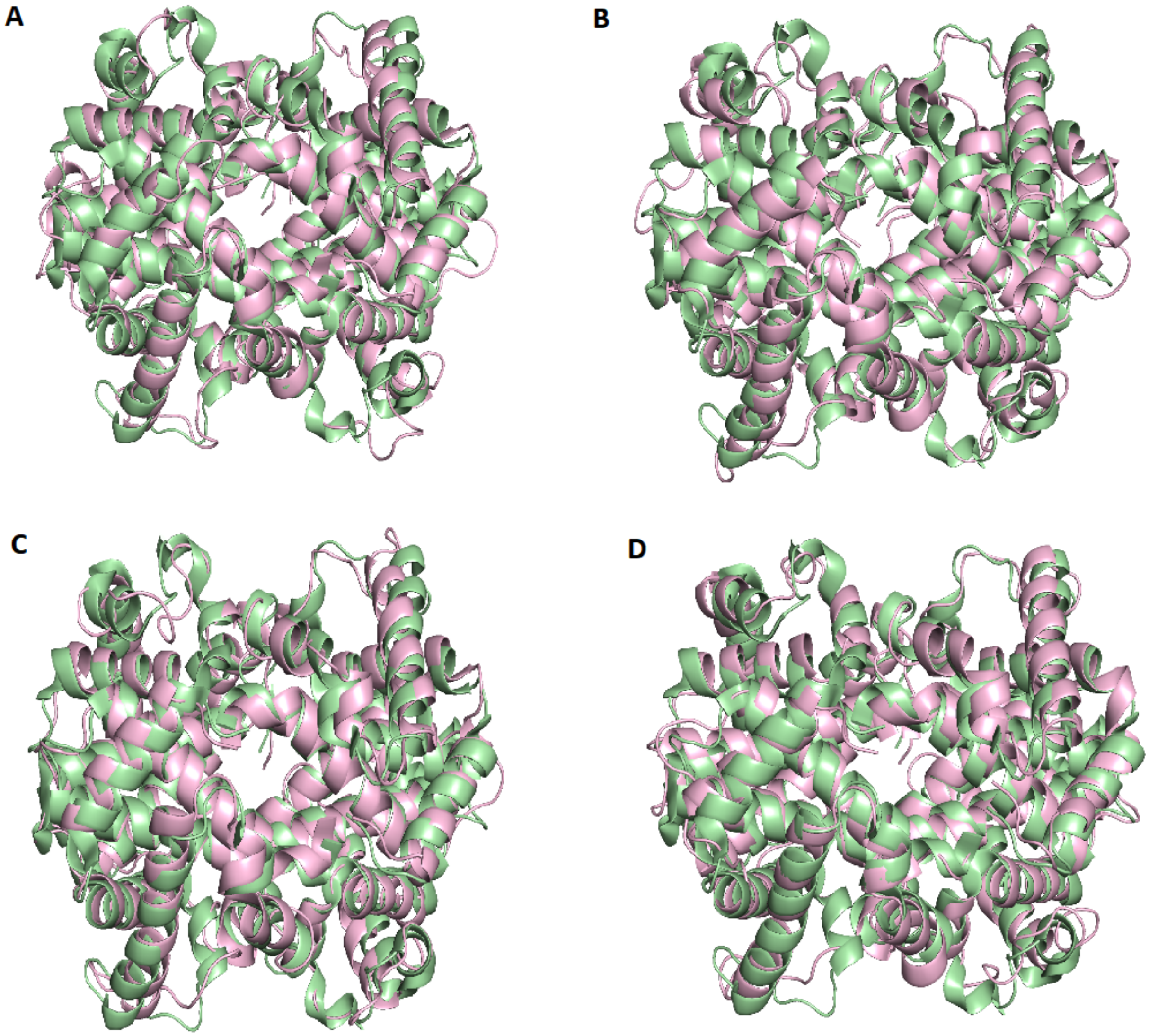

Figure 7. The effect of different temperature conditions on the structure of camel Hb. Crystal structure (PDB ID: 3GDJ) and simulated structure of Hb are shown in green and pink cartoon representation, respectively. A) Superimposition of the 3GDJ structure and the Hb structure (t=500 ns) simulated at 27 °C. B) Superimposition of the 3GDJ structure and the Hb structure (t=500 ns) simulated at 30 °C. C) Superimposition of the 3GDJ structure and the Hb structure (t=500 ns) simulated at 34 °C. D) Superimposition of the 3GDJ structure and the Hb structure (t=500 ns) simulated at 41 °C. Images were generated using the PyMOL Molecular Graphics System version 2.0 (http://www.pymol.org/pymol).

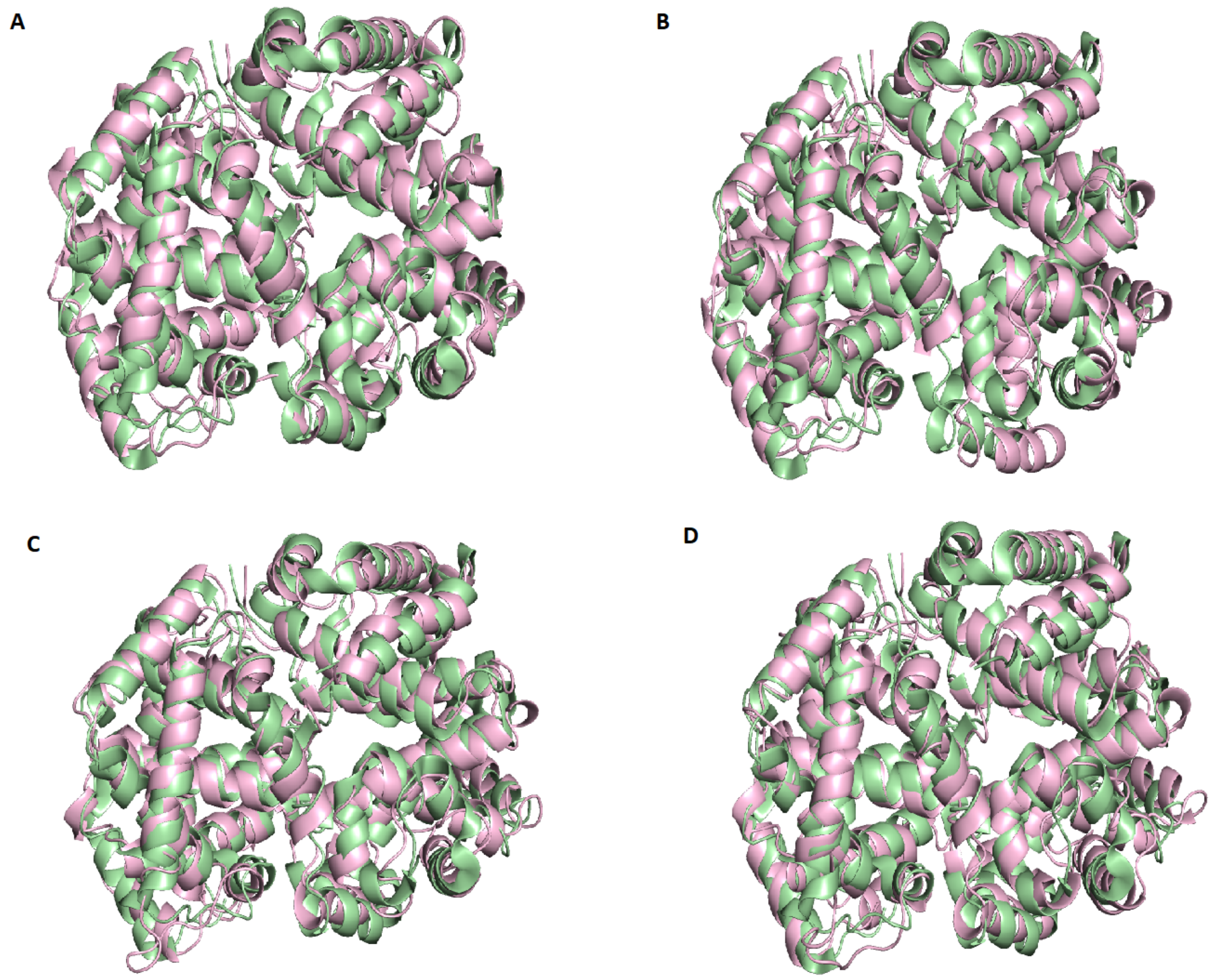

Figure 8. Effect of different temperature conditions on the structure of human Hb. Crystal structure (PDB ID: 1BBB) and simulated structure of Hb are shown in green and pink cartoon representation, respectively. A) Superimposition of the 1BBB structure and the Hb structure (t=500 ns) simulated at 27 °C. B) Superimposition of the 1BBB structure and the Hb structure (t=500 ns) simulated at 30 °C. C) Superimposition of the 1BBB structure and the Hb structure (t=500 ns) simulated at 34 °C. D) Superimposition of the 1BBB structure and the Hb structure (t=500 ns) simulated at 41 °C. Images were generated using the PyMOL Molecular Graphics System version 2.0 (http://www.pymol.org/pymol).
